# Stability analysis of a state-dependent delay differential equation for cell maturation: analytical and numerical methods

**DOI:** 10.1007/s00285-019-01357-0

**Published:** 2019-04-19

**Authors:** Philipp Getto, Mats Gyllenberg, Yukihiko Nakata, Francesca Scarabel

**Affiliations:** 1 Center for Dynamics, Technische Universität Dresden, 01062 Dresden, Germany; 2 Department of Mathematics and Statistics, University of Helsinki, 00014 Helsinki, Finland; 3 Department of Mathematical Sciences, Shimane University, Matsue, 690-8504 Japan; 4 Present Address: LIAM, Department of Mathematics and Statistics, York University, 4700 Keele St, Toronto, ON M3J 1P3 Canada; 5 CDLab, Department of Mathematics, Computer Science and Physics, University of Udine, Udine, Italy

**Keywords:** Characteristic equation, Pseudospectral, Linearised stability, Threshold-type delay, Stem cell, Progenitor phase, 65P30, 34K20, 37M20, 92C37, 92D25

## Abstract

We consider a mathematical model describing the maturation process of stem cells up to fully mature cells. The model is formulated as a differential equation with state-dependent delay, where maturity is described as a continuous variable. The maturation rate of cells may be regulated by the amount of mature cells and, moreover, it may depend on cell maturity: we investigate how the stability of equilibria is affected by the choice of the maturation rate. We show that the principle of linearised stability holds for this model, and develop some analytical methods for the investigation of characteristic equations for fixed delays. For a general maturation rate we resort to numerical methods and we extend the pseudospectral discretisation technique to approximate the state-dependent delay equation with a system of ordinary differential equations. This is the first application of the technique to nonlinear state-dependent delay equations, and currently the only method available for studying the stability of equilibria by means of established software packages for bifurcation analysis. The numerical method is validated on some cases when the maturation rate is independent of maturity and the model can be reformulated as a fixed-delay equation via a suitable time transformation. We exploit the analytical and numerical methods to investigate the stability boundary in parameter planes. Our study shows some drastic qualitative changes in the stability boundary under assumptions on the model parameters, which may have important biological implications.

## Introduction

Maturation of cells, from the stem cell phase up to the fully mature phase, is an essential, and yet not completely understood, phenomenon. It is particularly interesting to understand what are the processes that promote a healthy equilibrium population state, or homeostasis, and what are those that may induce destabilisation of the equilibrium and appearance of periodic behaviour. We remark that oscillations play an important role in stem cell population dynamics in relation to hematological disorders like cyclic neutropenia and periodic myelogenous leukemia, see for instance Mackey (1978), Bernard et al. (2003), Pujo-Menjouet et al. (2005), and Adimy and Crauste (2012).

In this paper we investigate a stem cell model where maturation is described as a continuous process, and we show that the stability properties of the positive equilibrium are crucially affected by the choice of the maturation speed. In particular, we prove that different choices of the maturation speed imply qualitative and quantitative changes in the regions of stability of the equilibrium in parameter planes. We achieve this by developing analytical and numerical methods for the analysis of equilibria and stability of structured population models.

**Fig. 1:**
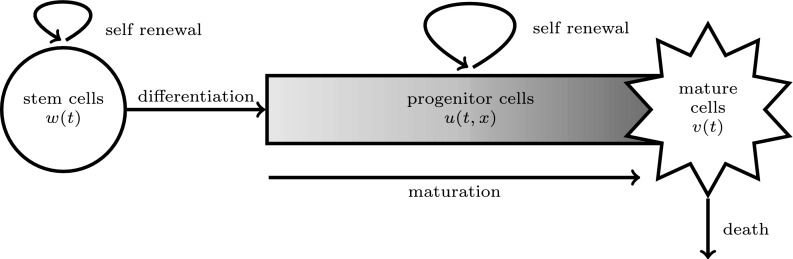
Schematic representation of the model: at time *t*, *w*(*t*) and *v*(*t*) denote the total amount of stem cells and mature cells, respectively; *u*(*t*, *x*) is the amount of progenitor cells with maturity $x\in [x_1,x_2]$. The processes are indicated in the figure

More precisely, the model is motivated by the blood cell production system and in particular by the production of white blood cells. It describes the process of division of cells by mitosis, their self-renewal, the differentiation of stem cells into progenitor cells (also called simply progenitors), and a continuous maturation process of the progenitors up to the transition into the mature cell compartment, see also Fig. 1. The mature cell population regulates the processes of division, self-renewal, differentiation and maturation. The development of progenitors, and in particular their self-renewal and maturation, depends on their maturity, which is described by a deterministically developing one-dimensional variable. In this way the progenitor population has continuous maturity structure. The latter is useful when it is not possible to divide the cell population into a finite number of discrete subpopulations. The continuous variable may represent a measurable quantity that evolves during maturation. This can be for instance age, size, or, as mentioned by Potten and Loeffler (1990) (to which we also refer for a short review of the biological terms), position in the tissue or “the weight of a specific protein per cell”. An advantage of keeping the model general, without specifying the maturity variable, is that it potentially applies to different cell types and laboratory measurements.

We note that a similar concept of continuous maturity structure in the context of blood cells is considered by Adimy et al. (2005). Hematopoietic stem cells are divided into a resting and a proliferating phase, and the duration of the proliferating phase is assumed to depend on the maturity at commitment. Adimy and Crauste (2009) introduced regulation of the processes by growth factors, which are for instance proteins like G-CSF or EPO, that are controlled by the amount of mature cells. Adimy et al. (2010) considered an age-structured model with fixed duration of the proliferating phase. We refer to Pujo-Menjouet (2016) for a recent review of models for blood cells. All the above-mentioned models focus on the stem cell phase, which they structure by maturity. Moreover, they assume that cells divide only at the end of the proliferating phase, and division is assumed to be symmetric: upon division, two identical cells are produced and enter immediately the resting phase.

We consider a model for cell maturation where stem cells are assumed to be unstructured, whereas the maturity structure is introduced in the progenitor compartment. Moreover, we include the case of asymmetric cell division: two daughter cells are not necessarily of the same type, but they may belong to different compartments (stem or progenitor). In particular, this is achieved with the concept of *fraction of self-renewal* of stem cells (see also *s*(*v*) in Table 1), representing the fraction of daughter cells that are themselves in the stem cell compartment. The complementary fraction of daughter cells is assumed to enter the progenitor compartment with the initial maturity level.

A model motivated by these assumptions, but including a finite number of compartments to describe the progenitor phase, was established by Marciniak-Czochra et al. (2009). The finite number of compartments can be replaced with continuous maturity structure, where a progenitor cell is assumed to either undergo maturation or divide and produce daughter cells at the same maturity stage. In the literature, this has been done in two ways (see also Sect. 2 for more details). The first approach is by means of a partial differential equation (PDE) of transport type for the progenitor cells, coupled with two ordinary differential equations (ODE) for, respectively, stem and (fully) mature cells. This was first done by Doumic et al. (2011). Alternatively one can formulate the system as a delay differential equation (DDE) for the mature cells, in which the delay is state-dependent, coupled with the ordinary differential equation for the stem cells. A first formulation as a state-dependent delay differential equation (SD-DDE) was derived by Alarcón et al. (2011). Here we consider a slightly adapted SD-DDE formulation derived by Getto and Waurick (2016) (see again Sect. 2).

By guaranteeing differentiability of the functional inducing the DDE and an application of a theoretical result for SD-DDE of Walther (2003), Getto and Waurick (2016) showed that the model is well posed and that a linear variational equation can be associated with the solutions. The stem cell model has a trivial equilibrium and a positive equilibrium, which emerges from the trivial in a transcritical bifurcation. Recall that the principle of linearised stability allows to determine the stability of the equilibrium with respect to perturbed initial conditions by locating the roots of a characteristic equation with respect to the imaginary axis.

The cell maturation model considered in this paper is an instance of a physiologically structured population model in the sense of Metz and Diekmann (1986) and Diekmann et al. (2001). Such models are often formulated as Volterra functional equations coupled with DDE, in which the latter do not feature state-dependent delays. The principle of linearised stability in this formulation was proven by Diekmann et al. (2007) for the case of finite delay, and by Diekmann and Gyllenberg (2012) for the case of infinite delay. Diekmann and Gyllenberg (2007) gave conditions for differentiability of the right-hand side of a Volterra functional equation describing size-dependent cannibalism, thus providing the key to linearised stability analysis. A size-structured consumer–resource model was formulated by Diekmann et al. (2010, 2017) as a Volterra functional equation, and the linearisation computed analytically. For a special case of the model, the differentiability assumption required in Diekmann et al. (2007) was proven by Diekmann and Korvasová (2016). We stress that, apart from these papers, very few proofs of linearised stability exist for physiologically structured population models. Therefore, there are substantive grounds for developing methods for a more in-depth stability analysis. Here were develop both analytical and numerical methods.

Regarding analytical methods, in this paper we combine the linear variational equation derived by Getto and Waurick (2016) with theoretical results on linearised stability for SD-DDE by Hartung et al. (2006) and Stumpf (2016), to guarantee that the principle of linearised stability holds for the stem cell model in the DDE formulation. Then, we use the linear variational equation to derive characteristic equations for arbitrary, trivial and positive equilibria, respectively. We use these to derive the following results. The trivial equilibrium is stable in absence of the positive one and unstable in its presence, and the positive equilibrium is stable upon emergence through a transcritical bifurcation. This result is in agreement with the analysis of the multi-compartment model of Stiehl and Marciniak-Czochra (2011) and with the analysis of the PDE model of Doumic et al. (2011). Regarding the positive equilibrium, we also prove that in the right half-plane there are no roots of the characteristic equation outside of a compact set. These results can be combined with an argument of Diekmann et al. (1995) to conclude that roots can enter the right half-plane only through a compact subset of the imaginary axis, and thus the positive equilibrium can destabilise only if the latter occurs.

By the previous arguments, it makes sense to analyse the characteristic equation for the positive equilibrium on the imaginary axis, but the complexity of the characteristic equation in full generality motivates us to focus on special scenarios. Here, we consider a simplified characteristic equation where the dependence on maturity and on mature cell population of the progenitor development, given by maturation and self-renewal, can be neglected. In the population dynamical formulation, this simplification would transform the SD-DDE to a DDE with fixed delay, meaning a fixed positive time delay between leaving the stem cell compartment and entering the mature cell compartment. For this case, we show that the positive equilibrium can destabilise by a pair of conjugate roots crossing into the right half-plane, which gives evidence for a Hopf bifurcation.

To visualise the results (both analytical and, later on, numerical), we consider specifications of the model ingredients taken or adapted from the available literature. We single out pairs of parameters and we distinguish, in the associated parameter plane, the regions in which the positive equilibrium is stable and the regions in which it is unstable. These regions are shown via a curve, representing the stability boundary, that is parametrised by the position of the roots on the imaginary axis. This approach was presented for a prototype characteristic equation by Diekmann et al. (1995) and elaborated for a somewhat more general characteristic equation by Alarcón et al. (2014), and later by Diekmann et al. (2016). The characteristic equation analysed here is yet slightly more involved and we show some qualitative features that are not shown in the former references: depending on a third parameter, the curve describing the stability boundary has a unique minimum or is monotonically increasing.

For the analysis of the stability boundaries in more general cases, we propose numerical methods. The perhaps most generally applicable method for stability analysis in continuous population dynamics is the pseudospectral discretisation approach. The approach was first applied for studying the stability of the zero solution of linear DDE with fixed delay by Breda et al. (2005, 2013), see also Breda et al. (2015b) for a review, and applied to the linearisation of a size-structured consumer–resource model by Breda et al. (2015a). The pseudospectral discretisation was then applied by Breda et al. (2016a) to nonlinear delay equations, including both differential and integro-differential equations with fixed delay, and later extended to equations with infinite delay by Gyllenberg et al. (2018). With this method, the nonlinear DDE is approximated with a system of ODE, whose properties can be studied numerically with the package matcont for matlab, a well-established tool for the numerical bifurcation analysis of ODE developed by Dhooge et al. (2003, 2008). Here, we extend the approach to the case of SD-DDE. This is done by exploiting the uniform bound to the state-dependent delay, which ensures that the states of the dynamical system associated with the DDE are defined on a fixed and bounded interval. The latter interval is then discretised with a finite number of points and the state of the system is projected in the finite-dimensional space of polynomials via the pseudospectral approximation method. To the best of our knowledge, this is the only technique that allows the user to study numerically the stability boundaries of DDE with history-dependent delay using available software packages.

Since this paper contains the first application of the pseudospectral approach to SD-DDE, here we put a strong emphasis on numerical validation, remarking that this benefits the credibility of both the methods, numerical and analytical, involved here. Validation can be carried out for the case of constant maturation speed mentioned above (for which the analytical methods can also be applied), and for a more general case of maturation speed independent of the current value of maturity, that we are going to describe below.

Suppose that the speed of maturation of progenitors is still allowed to depend on the mature cell population, but it is independent of the maturity of the progenitor cell. This is a mathematically motivated simplification which may apply to a biological scenario in which the maturation rate of a progenitor cells at any stage is regulated only by external growth factors, which we assume regulated only by the population of mature cells. In the formulation of the population model, this means that the delay is still state-dependent, but the state-dependence is somewhat more explicit. In this case, a suitable time transformation allows to rewrite the SD-DDE as an equation with fixed delay.

We mention that, for a scalar equation with threshold-type delay, an analogue time transformation was first considered by Smith (1992, 1993). More recently, the transformation has been successfully applied to study the dynamical and bifurcation properties of delay models describing complex structured ecological systems, for instance by McCauley et al. (2008), Nelson et al. (2013), Bjørnstad et al. (2016) and Nisbet (1997). Apart from these applications, we could not find in the literature a rigorous statement of the correspondence of solutions of the two systems and of the related stability properties. Here we define the transformation explicitly introducing a parametrised map, and we show rigorously the invariance of equilibria and stability properties.

We then remark that DDE with merely fixed delays can be studied numerically with the package dde-biftool for matlab, developed by Engelborghs et al. (2002). Like matcont for ODE, dde-biftool allows the user to perform for instance the one-parameter continuation of equilibria and periodic solutions, the computation of eigenvalues and multipliers, and the continuation of bifurcation points in two parameters. Hence, in the case of maturation speed independent of maturity, there are three numerical methods for computing stability boundaries at disposal: pseudospectral method (and matcont analysis) applied to the SD-DDE, pseudospectral method applied after the transformation to a fixed-delay DDE, and dde-biftool after the transformation. We stress moreover that, in the case of a fixed delay in the untransformed model, the stability boundaries are available analytically and can be compared with the numerically computed curves. We use all of the above methods to compute stability boundaries and find that, upon changing the method, differences in the visualised boundaries are not distinguishable by eye. These tests support the validity of the pseudospectral approximation applied to SD-DDE, and justify the application of the method for studying the stability boundaries of more general instances of the model for which, as already mentioned, no other software package is available.

The stability boundaries presented here are the first to be computed for this model. They are carried out for rate specifications taken from the literature from Marciniak-Czochra et al. (2009), Doumic et al. (2011) and Stiehl et al. (2014). The specifications include different regulation mechanisms of the stem cell processes, like division or self-renewal, by the mature cell population. As for the progenitor maturation rate, for which we could not find an established formulation, we considered a new formulation that can incorporate different types of dependence on both maturity and mature cells. Of potential biological interest are some rather drastic changes in the curves upon changing these regulation modes. A cell biological interpretation of these changes and laboratory validation are some of the future perspectives of this research.

The paper is structured as follows. Section 2 introduces the model, see Eqs. (2.5)–(2.8). In Sect. 3 we recall some theoretical results about existence and uniqueness of solutions and the principle of linearised stability for equilibria. In Sect. 4 we compute the equilibria of the general model and the corresponding characteristic equations; we give conditions for the existence of a positive equilibrium which destabilises the trivial, and we show that the positive equilibrium is stable upon emergence. In Sect. 5 we restrict to a particular instance of Eqs. (2.5)–(2.8) with fixed delay; we give analytical formulas for the stability boundary of the positive equilibrium and we describe how the boundary changes qualitatively when the maturation rate changes. In Sect. 6 we consider the case of a maturation rate independent of maturity: we introduce a time transformation to define an equation with fixed delay with the same equilibria and the same stability properties. In Sect. 7, we explain how to approximate SD-DDE of the type (2.5)–(2.8) with the pseudospectral discretisation technique, and we specify the approximating system of ODE. Finally, in Sect. 8 we consider different assumptions on the maturation rate and we investigate numerically the effect on the stability boundary of the positive equilibrium. The proofs of the mathematical results of Sects. 3–6 are collected in the “Appendix”. We have tried to structure and highlight the paper such that each reader, inclined either to biological, computational or analytical aspects, should be able to find the relevant topics, with no need of going deeply into details about the other disciplines. In particular, we tried to present the main results and Sect. 9, which also contains a discussion of the potential biological relevance of the results, in a rather self-contained way.

## The model

We consider a model for cell maturation studied in PDE formulation by Doumic et al. (2011) and in DDE formulation by Getto and Waurick (2016). A schematic illustration of the model is given in Fig. 1.

Cells are divided into stem cells, progenitor cells and fully mature cells. Stem cells and fully mature cells are unstructured in the sense that only their total amount, *w*(*t*) and *v*(*t*), respectively, affect the population dynamics. Progenitor cells, on the other hand, are structured by a one dimensional quantity denoted by *x* and taking on values in the finite interval $[x_1,x_2]$. We let *u*(*t*, *x*) denote the density of progenitor cells with maturity *x* at time *t*.

We assume that the maturation process, including stem cell self-renewal and division, is subject to regulation by the mature cells *v* and by those only. In the terminology of Diekmann, Metz and collaborators [Metz and Diekmann (1986), Diekmann et al. (2001, 2003, submitted)], *v* plays the role of the environmental condition.

Let *q*(*v*) denotes the net growth rate of the stem cell population. This rate incorporates division, self-renewal, differentiation, which means transition to the progenitor compartment, and apoptosis, or mortality, of cells. The dynamics of the stem cells is given by the ordinary differential equation

2.1 $$\begin{aligned} w'(t)=q(v(t))w(t). \end{aligned}$$

Next, let $g(x,v)>0$ and *d*(*x*, *v*) be the maturation rate and net production rate, respectively, of progenitor cells. The rate *d* captures both self-renewal and decay of progenitor cells. The density *u*(*t*, *x*) of progenitor cells satisfies the PDE

2.2 $$\begin{aligned} \frac{\partial }{\partial t}u(t,x) + \frac{\partial }{\partial x}(g(x,v(t))u(t,x)) = d(x,v(t))u(t,x). \end{aligned}$$

At any time the total outflow from the stem cell compartment equals the inflow into the progenitor cell compartment. Therefore the PDE (2.2) must be supplemented by the following boundary condition:

2.3 $$\begin{aligned} g(x_1,v(t)) u(t,x_1) = \gamma (v(t))w(t). \end{aligned}$$

Finally, progenitor cells are assumed to become fully mature upon reaching maturity $x_2$ and this leads to the following equation for the fully mature cells:

2.4 $$\begin{aligned} v'(t) = g(x_2,v(t))u(t,x_2) -\mu v(t), \end{aligned}$$

where $\mu$ is the per capita death rate of fully mature cells.

The system (2.1)–(2.4) specifies a physiologically structured population model. Observe that if *v*(*t*) is assumed to be a known function of time, the system (2.1)–(2.3) is a time-dependent *linear* system for *w* and *u*. The *feedback* described by (2.4) makes the full system into a nonlinear autonomous system.

The progenitor cell compartment can be eliminated from the system (2.1)–(2.4) by careful book-keeping. Notice that the progenitor cells reaching full maturity at $x=x_2$ at time *t* are the stem cells that differentiated and became progenitor cells with maturity $x=x_1$ at some time $t - \tau$ earlier plus those who have been born on the way due to self-renewal minus those who have died. Because the maturation rate *g* depends on the current density of fully mature cells, the maturation delay $\tau$ depends on the *history*$v_t$ of the fully mature cells, where we have used the standard notation

$$\begin{aligned} x_t(s):=x(t+s),\quad s<0, \end{aligned}$$

if a function *x* is defined in $t+s$.

To define $\tau (v_t)$ we first define the function $y(\cdot ,v_t)$ as the unique solution of the initial value problem

2.5 $$\begin{aligned} \begin{aligned} y'(s)&=-g(y(s),v_t(-s)), \quad s>0,\\ y(0)&= x_2, \end{aligned} \end{aligned}$$

given the history $v_t$ of the fully mature cells. Note that $y(s,v_t)$ is the maturity of a progenitor cell *s* time units before it reaches full maturity at time *t*, that is, its maturity at time $t-s$.

Because the maturation rate *g* is assumed to be positive, the function $y(\cdot ,v_t)$ is monotone and therefore for any given history $v_t$, the equation

2.6 $$\begin{aligned} y(\tau ,v_t)=x_1, \end{aligned}$$

has a unique solution $\tau (v_t)$, which is the time it took for a progenitor cell to mature from $x=x_1$ to $x=x_2$, given that it reached $x=x_2$ at time *t*.

The book-keeping alluded to above amounts to integrating the PDE (2.2) along characteristics (Metz and Diekmann 1986). This was done by Getto and Waurick (2016). Substituting the resulting expression for $u(t,x_2)$ into (2.4) they obtained

2.7 $$\begin{aligned} w'(t)= & {} q(v(t))w(t), \end{aligned}$$

2.8 $$\begin{aligned} v'(t)= & {} \frac{\gamma (v(t-\tau (v_t)))g(x_2,v(t))w(t-\tau (v_t))}{g(x_1,v(t-\tau (v_t)))}\mathop {}\!\mathrm {e}^{\int _0^{\tau (v_t)}(d-D_1g)(y(s,v_t),v(t-s))\mathop {}\!\mathrm {d}s}\nonumber \\&-\,\mu v(t). \end{aligned}$$

Here and in the rest of the paper, the notation $D_j$ denotes the derivative of a function with respect to its *j*-th argument.

The Eqs. (2.7) and (2.8) with *y* and $\tau$ given by (2.5) and (2.6), respectively, constitute the model which will be studied in the current paper.

Equations (2.7) and (2.8) consist of an ODE coupled with a differential equation with state-dependent and distributed delay (DDE) in which the state-dependent delay is defined via a threshold condition. We remark that a more explicit derivation of the DDE directly from first principles was attempted by the first author in a paper by Alarcón et al. (2011). However, that derivation erroneously missed the integral of $D_1g$ in the exponent (the *dilation factor*, see Metz and Diekmann (1986)), as well as the ratio $g(x_2,v(t))/g(x_1,v(t-\tau (v_t)))$, which comes from the equation of fluxes at the boundaries of the maturation interval, taking into account that the maturation speeds differ at the boundaries $x_1$ and $x_2$.

For a more in-depth analysis we will use the specifications for *q* and $\gamma$ as given in Table 1. These ingredients were derived by Marciniak-Czochra et al. (2009) for a multi-compartment model describing hematopoietic stem cells producing leukocytes, and later considered by Getto and Marciniak-Czochra (2015) for the multi-compartment model as well as for the present model. They are based on the assumption that the individual stem cell division rate ($d_w(v)$) and the fraction of self-renewal (*s*(*v*)) are regulated by a single external feedback mechanism through some signalling molecules called “cytokines”. The signal may either enhance the division rate or the fraction of self-renewal. The mechanistic derivation of the signal intensity (which justifies the form of the rates *s*(*v*) and $d_w(v)$ in Table 1) was obtained by Marciniak-Czochra et al. (2009) through a quasi-steady state approximation. In this paper, we either consider a generic progenitor production rate *d* or neglect progenitor production by assuming $d\equiv 0$. For *g* we will consider various choices that will be given below.

In the model of Marciniak-Czochra et al. (2009) stem cells are still described by (2.7), whereas progenitors are divided into a discrete number of compartments in which cells can divide, self-renew and differentiate (similarly as stem cells). Accordingly, the rates of inflow and net production of progenitor cells are described by rates with the same algebraic construction as the rate of inflow and net growth rate in Table 1, but possibly for different parameter values. Alarcón et al. (2011) and Doumic et al. (2011) replaced the discrete number of progenitor compartments by one progenitor compartment featuring continuous maturity structure as in (2.5) and (2.6). The dynamics within this compartment can be described by a classical transport equation. If the rates are specified as in Table 1, the model assumptions are equivalent to those of Doumic et al. (2011), and the results can be compared to this paper.

**Table 1:** Specifications for stem cell rates, from Marciniak-Czochra et al. (2009) and Getto and Marciniak-Czochra (2015)

Description	Function
Net growth rate	$q(v)=\left[ 2s(v)-1\right] d_w(v)-\mu _w$
Rate of inflow into progenitors	$\gamma (v) = 2\left[ 1-s(v)\right] d_w(v)$
Division rate	$d_w(v) = \frac{p}{1+k_p v}$
Fraction of self-renewal	$s(v)=\frac{a}{1+k_a v}$

The parameter $\mu _w$ is the stem cell mortality rate. The parameters $a,p,k_a,k_p$ are nonnegative, with $a>0.5$

## Linearised stability theorems

We show that the model can be analysed using results for a general class of DDE of the form

3.1 $$\begin{aligned} x'(t)=f(x_t), \end{aligned}$$

where $f:{\mathcal {O}} \rightarrow {\mathbb {R}}^n$ is defined on a suitable open subset ${\mathcal {O}} \subset C^1([-h,0],{\mathbb {R}}^n)$ for an appropriately chosen $h\in (0,\infty )$. The model (2.5)–(2.8) can obviously be written as an equation of the above type if we set $f=F$ and define

$$\begin{aligned} F_1(\varphi ,\psi )= & {} q(\psi (0))\varphi (0)\\ F_2(\varphi ,\psi )= & {} \frac{\gamma (\psi (-\tau (\psi )))g(x_2,\psi (0))\varphi (-\tau (\psi ))}{g(x_1,\psi (-\tau (\psi )))} \mathop {}\!\mathrm {e}^{\int _0^{\tau (\psi )}(d-D_1g)(y(s,\psi ),\psi (-s))\mathop {}\!\mathrm {d}s}\\&-\,\mu \psi (0). \end{aligned}$$

for $(\phi ,\psi )\in \mathcal{O}\subset C^1([-h,0],{\mathbb {R}}^2)$, that will be specified below. We will consider two equilibrium states and the long-term behaviour of solutions starting closeby. To this aim we present two results, which refer to the class of DDE (3.1) and to the stem cell model (2.5)–(2.8), respectively. These state that, under certain conditions, the well-known *principle of linearised stability* holds. The proofs, along with associated results on existence and uniqueness of solutions for the respective systems, follow from a compilation of results from the literature that will be given in the following. We refer to this literature also for the mathematical details that we do not present here. Following Hartung et al. (2006), we say that *f* satisfies property (S) if the following hold:

1. *f* is continuously differentiable;
2. each derivative $Df(\phi )$ extends to a linear map $D_ef(\phi )$ defined on $C([-h,0],{\mathbb {R}}^n)$;
3. the map $(\phi ,\chi ) \mapsto D_ef(\phi )\chi$ is continuous.

Moreover, we additionally introduce for *f* the following property (whose name stands for “strongly Lipschitz, uniformly on bounded sets”)

(sLb) for any bounded set $B\subset {\mathcal {O}}$ there exists some $L_B>0$ such that
  $$\begin{aligned} |f(\phi _1)-f(\phi _2)| \le L_B \Vert \phi _1-\phi _2\Vert , \quad \text { for all } \phi _1,\phi _2 \in B. \end{aligned}$$

The latter property is used for instance by Walther (2003). We define a function $e_z$ by introducing the notation

$$\begin{aligned} e_z(\theta ):=\mathop {}\!\mathrm {e}^{z\theta }. \end{aligned}$$

### Theorem 3.1

Suppose that *f* satisfies conditions (S) and (sLb). Then the DDE (3.1) is well posed (in the sense of Hartung et al. (2006)). If additionally ${\overline{x}}$ is a constant function satisfying $f({\overline{x}})=0$, then ${\overline{x}}$ is locally asymptotically stable if all the roots $z\in {\mathbb {C}}$ of the characteristic equation

3.2 $$\begin{aligned} \det (zI-Df({\overline{x}})e_z)=0 \end{aligned}$$

have negative real part, and unstable if the equation has a root with positive real part.

The proof essentially follows from the works of Diekmann et al. (1995), Walther (2003), Hartung et al. (2006), Stumpf (2016), and Getto and Waurick (2016). In the “Appendix”, in “Proof of Theorem 3.1”, we present a more precise argumentation.

We now introduce some assumptions on the model ingredients that are used to prove the principle of linearised stability for the model (2.5)–(2.8). We denote open balls by $B(x_0,b):=\{x\in {\mathbb {R}}:|x-x_0|<b\}$ for some $x_0\in {\mathbb {R}}$ and some $b>0$. First, we assume that *g* satisfies property (G) by Getto and Waurick (2016), that we recall here for convenience: there exist $b,K,\varepsilon \in {\mathbb {R}}$ and open intervals *I*, *J*, with $0 \in I$, such that

1. $$B(x_2,b) \subset J$$ and $$g :J\times I \rightarrow {\mathbb {R}}$$ is $$C^1$$;
2. $D_1g(x,y)$ is bounded on $B(x_2,b)\times I$;
3. $$0<\varepsilon \le g(x,y) \le K$$ on $$B(x_2,b)\times I$$ and $$x_2-x_1 \in (0,\frac{b}{K}\varepsilon )$$.

With these assumptions, *h* can be defined as $h:=\frac{b}{K}$.

Finally, we make the following additional assumptions on the model ingredients:

- $d, D_1g:J\times I \rightarrow {\mathbb {R}}$ are $C^1$, bounded on $\overline{B(x_2,b)}\times I$, and
  $$\begin{aligned} \sup _{(x,y)\in \overline{B(x_2,b)}\times I} |D_1g(x,y)| < \frac{K}{b}; \end{aligned}$$
- $D_2g$, $D_i d$ and $D_i D_1 g$, $i=1,2,$ are bounded on $B(x_2,b)\times A$, whenever $A\subset I$ is bounded;
- $\gamma :I \rightarrow {\mathbb {R}}_+$ and $q:I \rightarrow {\mathbb {R}}$ are continuously differentiable, and Lipschitz on bounded sets.

Under these conditions, Getto and Waurick (2016) showed (see Theorem 1.13) that *F* satisfies (S) and (sLb). Hence Theorem 3.1 can be applied, and this immediately yields the following result.

### Corollary 3.2

Suppose that *g* satisfies property (G), $\mu$ is a positive parameter, and *g*, *d*, $\gamma$ and *q* satisfy assumptions (a)–(c). Then the stem cell model (2.5)–(2.8) is well posed (in the sense of Hartung et al. (2006)) and the solutions exist for all times. If additionally (*w*, *v*) is a constant function satisfying $F(w,v)=0$, then (*w*, *v*) is locally asymptotically stable if all the roots $z\in {\mathbb {C}}$ of

3.3 $$\begin{aligned} \det (zI-DF(w,v)e_z)=0 \end{aligned}$$

have negative real part, and unstable if the equation has a root with positive real part.

For completeness, we repeat here some results from Getto and Waurick (2016) that will be useful later on. For ${\mathcal {O}}:= C^1([-h,0],{\mathbb {R}})\times C^1([-h,0],I)$, the set

3.4 $$\begin{aligned} X = X(F) := \{(\varphi ,\psi )\in {\mathcal {O}} :(\varphi ,\psi )'(0)=F(\varphi ,\psi )\} \end{aligned}$$

is a $C^1$-submanifold of $C^1([-h,0],{\mathbb {R}}^2)$. As clear from the definition, *X* contains the segments of any solution after a certain finite time, including the constant functions satisfying the equilibrium conditions. For this reason *X* is called *solution manifold*. From (S) and (sLb) it follows that the delay functional is well defined by (2.5) and (2.6) and satisfies

3.5 $$\begin{aligned} \tau (\psi ) \in (0,h) \end{aligned}$$

for all $(\varphi ,\psi )\in X$. The system (2.5)–(2.8) supplemented with the initial condition

3.6 $$\begin{aligned} (w,v)_0=(\varphi ,\psi ), \quad (\varphi ,\psi ) \in X, \end{aligned}$$

is well posed, and a differentiable semiflow can be associated. Moreover, Getto and Waurick (2016) computed an expression for the derivative *DF*, which we will use in the present manuscript to analyse (3.3).

## Equilibrium and stability analysis

### Equilibria

Let us denote by (*w*, *v*) an equilibrium solution. If $w=0$ there is exactly one equilibrium, the trivial equilibrium $(w,v)=(0,0)$. If $w\ne 0$ the equilibrium conditions are

4.1 $$\begin{aligned} q(v)=0\;\; \mathrm{and}\;\; w\frac{\gamma (v)g(x_2,v)}{g(x_1,v)}\mathop {}\!\mathrm {e}^{\int _0^{\tau (v)}(d-D_1g)(y(s,v),v)\mathop {}\!\mathrm {d}s}=\mu v. \end{aligned}$$

We assume that, on $[0,\infty )$, the rate *q* decreases monotonically to a negative value. Then there exists a unique positive equilibrium if and only if $q(0)>0$. Thus, if we interpret *q*(0) as a bifurcation parameter, there is a transcritical bifurcation at $q(0)=0$.

### Characteristic equations

We now present some results that will be used to compute and analyse the characteristic equation. All the proofs are collected in the “Appendix”. Given a function *f* defined on $[0,\tau ]$, we denote by ${\widehat{f}}$ its Laplace transform, defined by

$$\begin{aligned} {\widehat{f}}(z):=\int _0^{\tau } f(t)\mathop {}\!\mathrm {e}^{-z t}\mathop {}\!\mathrm {d}t, \quad \text {for } z\in {\mathbb {C}}. \end{aligned}$$

Let us denote by (*w*, *v*) the *positive* equilibrium. We will also use the equilibrium conditions (4.1), the latter of which allows to eliminate *w*.

#### Lemma 4.1

For the zero equilibrium the characteristic equation reads

$$\begin{aligned} (z-q(0))(z+\mu )=0. \end{aligned}$$

If $q(0)>0$, for the positive equilibrium the characteristic equation is

4.2 $$\begin{aligned} \chi (v,z)=0, \end{aligned}$$

where

4.3 $$\begin{aligned} \chi (v,z)&:=z\widehat{k(v)}(z)+\mu v \mathop {}\!\mathrm {e}^{-\tau (v)z}\left[ z\left( \frac{\gamma '}{\gamma }(v)-\frac{D_2g}{g}(x_1,v)\right) +q'(v)\right] -z^2 \nonumber \\&\quad +\left( v\frac{D_2g}{g}(x_2,v)-1\right) \mu z, \nonumber \\ k(v)(t)&:=\mu v \bigg [D_2(d-D_1g)(y(t,v),v) \nonumber \\&- \frac{d-D_1g}{g}(x_1,v) \mathop {}\!\mathrm {e}^{-\int _t^{\tau (v)}D_1g(y(\theta ,v),v)\mathop {}\!\mathrm {d}\theta }D_2g(y(t,v),v) \nonumber \\&- D_2g(y(t,v),v)\int _t^{\tau (v)}D_1(d-D_1g)(y(\sigma ,v),v)\mathop {}\!\mathrm {e}^{-\int _t^{\sigma }D_1g(y(\theta ,v),v)\mathop {}\!\mathrm {d}\theta }\mathop {}\!\mathrm {d}\sigma \bigg ]. \end{aligned}$$

### Exchange of stability at the transcritical bifurcation and a priori bounds

We first conclude that the positive equilibrium destabilises the trivial one.

#### Corollary 4.2

The roots of the characteristic equation for the trivial equilibrium are *q*(0) and $-\mu$. Thus the trivial equilibrium is locally asymptotically stable if $q(0)<0$ and unstable if $q(0)>0$.

We next show that, if the positive equilibrium is sufficiently small, then the characteristic equation has exactly one root and this root is negative.

#### Lemma 4.3

Suppose that $q(0)>0$ and consider (4.2). Then $\chi (0,0)=0$, *f* is analytic, and there exists a $C^1$-function $v\mapsto z(v)$, defined in a neighbourhood of zero, with $z(0)=0$, such that, in a neighbourhood of (0, 0), (*v*, *z*(*v*)) is the unique solution. Moreover

$$\begin{aligned} z'(0)=q'(0)<0. \end{aligned}$$

Hence, by our earlier assumptions on *q*, if *q*(0) is sufficiently small then *v* is small enough for the corresponding unique root *z*(*v*) to be negative. Hence, the positive equilibrium is stable upon emergence. In summary we have shown an exchange of stability at the transcritical bifurcation.

We have shown that for *q*(0) sufficiently small the characteristic equation has no roots in the right half-plane. Our next question is whether the positive equilibrium can destabilise if *q*(0) moves further away from zero. In the following result we prove some a priori bounds for the roots. The result will be used to establish a maximal region of stability in parameter spaces.

#### Lemma 4.4

Suppose that $q(0)>0$ and consider (4.2). Then there exists a *K* such that, for every root *z* with $\mathop {}\!\mathrm {Re}\,z>0$, one has $|z|\le K$.

We conclude this section by presenting the specification of the characteristic equation in some particular cases. If $D_1g\equiv 0$, we omit the first argument in the notation of *g* and write *g*(*v*) and $g'(v)$ instead of *g*(*x*, *v*) and $D_2g(x,v)$ respectively. Further down we consider the case where *g* is a constant function, the value of which we also denote by *g*. We will use similar notation for $\tau$. We then get the following result.

#### Lemma 4.5

(a) If $D_1g\equiv 0$, the characteristic equation becomes

4.4 $$\begin{aligned}&z\widehat{k(v)}(z)+\mu v\left[ z\left( \frac{\gamma '}{\gamma }-\frac{g'}{g}\right) (v)+q'(v)\right] \mathop {}\!\mathrm {e}^{-\tau (v)z}\nonumber \\&\quad -\,z^2+\left( v\frac{g'}{g}(v)-1\right) \mu z=0, \end{aligned}$$

with

4.5 $$\begin{aligned} k(v)(t)=\mu v\, \big (D_2d(y(t,v),v)-\frac{g'}{g}(v)d(y(t,v),v)\big ). \end{aligned}$$

(b) If moreover $D_2d\equiv 0$, $D_2g\equiv 0$, it becomes

4.6 $$\begin{aligned}&\mu v\left[ \frac{\gamma '}{\gamma }(v)z+q'(v)\right] \mathop {}\!\mathrm {e}^{-\tau z}-z^2-\mu z=0,\;\;\tau =\frac{x_2-x_1}{g}. \end{aligned}$$

(c) If moreover *q* and $\gamma$ are as specified in Table 1, it becomes

4.7 $$\begin{aligned} \mu v\left[ \Big (\frac{d_w'}{d_w}(v)-\frac{s'(v)}{1-s(v)}\Big )z+2(s'd_w)(v)+d_w'(v)(2s(v)-1)\right] \mathop {}\!\mathrm {e}^{-\tau z}-z^2-\mu z=0. \nonumber \\ \end{aligned}$$

(d) If moreover $s\equiv a$ for some parameter $a>0.5$ ($k_a=0$ in Table 1), it becomes

4.8 $$\begin{aligned}&\frac{d_w'}{d_w}(v)\mu v(z+\mu _w)\mathop {}\!\mathrm {e}^{-\tau z}-z^2-\mu z=0. \end{aligned}$$

(e) If moreover $d_w$ is as specified in Table 1, it becomes

4.9 $$\begin{aligned}&\left[ 1-\frac{\mu _w}{(2a-1)p}\right] \mu (z+\mu _w)\mathop {}\!\mathrm {e}^{-z\tau }+z^2+\mu z=0. \end{aligned}$$

Note that, after the specifications, the existence condition $q(0)>0$ for the positive equilibrium can be translated to

4.10 $$\begin{aligned} p>\mu _w/(2a-1). \end{aligned}$$

Then the existence region in the $(\mu ,p)$-plane is the region above the line $p=\mu _w/(2a-1)$. Upon crossing the line from below, a transcritical bifurcation with exchange of stability occur, see Fig. 4.

## Determination and analysis of the stability boundary for the positive equilibrium for an example with fixed delay

### Destabilisation of the positive equilibrium

In this section we restrict to the case with fixed delay specified by the conditions of Lemma 4.5(e), and we analyse more in detail the stability boundary of the positive equilibrium. Multiplying (4.9) by $\tau ^2$, the following is proven.

#### Lemma 5.1

If *p* and $\mu$ are free parameters and the remaining ones are fixed, (4.9) can be expressed as

5.1 $$\begin{aligned} rm(\lambda +\eta ) \mathop {}\!\mathrm {e}^{-\lambda }+\lambda ^2+m\lambda =0, \end{aligned}$$

where we introduced the increasing functions

$$\begin{aligned} r(p):=1-\frac{\mu _w}{(2a-1)p},\;\;m(\mu ):=\mu \tau , \end{aligned}$$

a scaled stem cell mortality $\eta :=\mu _w\tau$, and a scaled complex variable $\lambda =z\tau$.

For $\lambda =\nu +i\omega$ the characteristic Eq. (5.1) can be written as the two real equations

$$\begin{aligned}&H_j(m,r,\nu ,\omega )=0,\;\;j=1,2,\;\;\mathrm{where}\\&H_1(m,r,\nu ,\omega ):=\nu ^2-\omega ^2+m\nu +mr\mathop {}\!\mathrm {e}^{-\nu }[(\nu +\eta )\cos \omega +\omega \sin \omega ],\\&H_2(m,r,\nu ,\omega ):=2\nu \omega +m\omega +mr\mathop {}\!\mathrm {e}^{-\nu }[\omega \cos \omega -(\nu +\eta )\sin \omega ]. \end{aligned}$$

Substituting $\nu =0$ and solving with respect to *r* and *m*, we get

5.2 $$\begin{aligned} r&=\frac{\omega }{\eta \sin \omega -\omega \cos \omega }, \end{aligned}$$

5.3 $$\begin{aligned} m&=\omega \frac{\eta \sin \omega -\omega \cos \omega }{\omega \sin \omega +\eta \cos \omega }. \end{aligned}$$

**Fig. 2:**
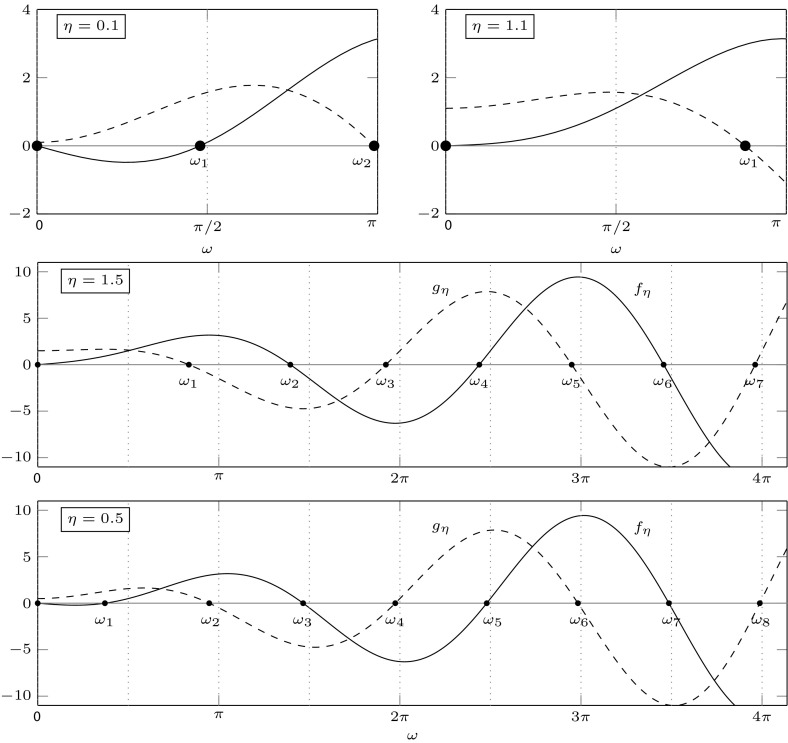
Graphs of $f_\eta$ (solid) and $g_\eta$ (dashed) for different values of $\eta$

**Fig. 3:**
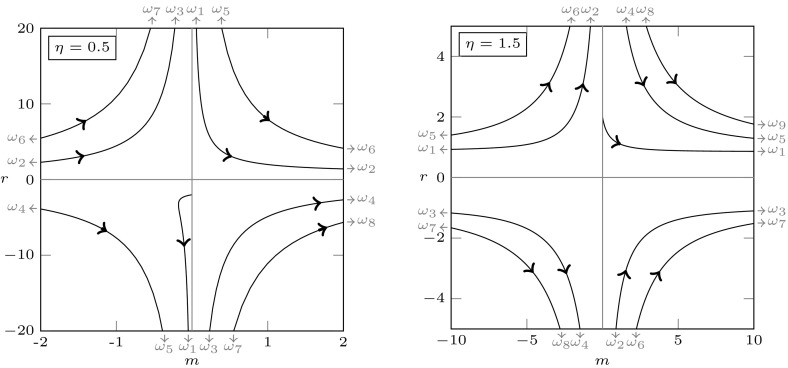
Graphs of $(m,r)(\omega )$ for $\eta =0.5$ and $\eta =1.5$. Existence boundary and region correspond to positive vertical axis and positive quadrant, respectively

Note that we should take care of the singularities of (5.2) and (5.3): we do this in the following result, together with a more complete analysis of the functions defining the denominators. For a visualisation of the following result, see Figs. 2 and 3. In the (*m*, *r*)-plane displayed in Fig. 3, the existence boundary corresponds to the positive vertical axis and the existence region to the positive quadrant.

#### Lemma 5.2

For $f_\eta (\omega ):=\eta \sin \omega -\omega \cos \omega$ and $g_\eta (\omega ):=\omega \sin \omega +\eta \cos \omega$ the following hold.

- For $\eta <1$ and $k=0,1,\dots$, one has
  (i) $f_\eta (2k\pi )=-2k\pi$ and $f_\eta '(2 k\pi )=\eta -1<0$; in every $(2k\pi ,2k\pi +\pi /2)$ there exists a unique zero; $f_\eta (2 k \pi +\pi /2)=\eta$; $f_\eta >0$ and $f_\eta '>0$ on $(\pi /2,\pi )+2k\pi$; in every $(\pi ,3\pi /2)+2k\pi$ there exists a unique zero; $f_\eta (3\pi /2+2k\pi )=-\eta$ and $f_\eta <0$ and $f'_\eta <0$ on $(3\pi /2,2\pi )+2 k\pi$;
  (ii) $g_\eta (2k\pi )=\eta$; $g_\eta '>0$ on $(0,\pi /2)+2k\pi$; $g_\eta (\pi /2+2k\pi )=\pi /2+2k\pi$; $g_\eta$ has a unique zero in each $(\pi /2,\pi )+2k\pi$; $g_\eta (\pi +2k\pi )=-\eta <0$; $g'_\eta <0$ on $(\pi ,3\pi /2)+2k\pi$, hence there are no zeros in these intervals. There exists a unique zero in every $(3\pi /2,2\pi )+2k\pi$.
  For $$\eta \ge 1$$,
  (iii) $f_\eta (0)=0$; $f_\eta$ has no zeros in $(0,\frac{\pi }{2}]$, and exactly one zero in every $(0,\frac{\pi }{2})+2k\pi$, $k=1,2,\dots$. Moreover, for every $k=0,1,\dots$, one has $f_\eta (\frac{\pi }{2}+2k\pi )=\eta$, ${{\,\mathrm{sgn}\,}}f_\eta =1$ on $[\frac{\pi }{2},\pi ]+2k\pi$, there exists a unique zero in $(\pi ,\frac{3\pi }{2})+2k\pi$, $f_\eta (\frac{3\pi }{2}+2k\pi )=-\eta$ and ${{\,\mathrm{sgn}\,}}f_\eta =-1$ on $[\frac{3\pi }{2},2\pi ]+2k\pi$;
  (iv) for $k=0,1,\dots$ one has $g_\eta (2k\pi )=\eta$ and $g_\eta$ has no zeros in $[0,\frac{\pi }{2}]$, neither in $[\pi ,\frac{3\pi }{2}]+2k\pi$, a unique zero in each $(\frac{\pi }{2},\pi )+2k\pi$ and in each $(\frac{3\pi }{2},2\pi )+2k\pi$.
- The function $\omega \mapsto (m,r)(\omega )$ has exactly the singularities $\omega _0<\omega _1<\dots$, where $\omega _0=0$ and, for $j=1,2,\dots$, the $\omega _j$ are the ordered remaining zeros of either $f_\eta$ or $g_\eta$ as determined in (a). In particular, for $j=1,2,\dots$, one has $\omega _j\in (\frac{\pi }{2}, \pi )+(j-1)\frac{\pi }{2}$ for $\eta \ge 1$ and $\omega _j\in (0, \frac{\pi }{2})+(j-1)\frac{\pi }{2}$ for $\eta <1$.
- For $\eta <1$ the function $f_\eta$ is increasing on $[\omega _1,\omega _2]$.

It follows from Proposition XI 2.13 of Diekmann et al. (1995) that, when crossing the curve $\omega \mapsto (m,r)(\omega )$ from right—in the sense of increasing $\omega$—to left, two complex conjugate roots cross from the left to the right half-plane, provided that the determinant in the following lemma is negative.

#### Lemma 5.3

One has $$ \det (\partial _i H_j(m,r,0,\omega ))_{1\le i,j\le 2}=-\omega m(\eta \cos \omega +\omega \sin \omega ). $$

We have shown that, in a right neighbourhood of the transcritical bifurcation point, the positive equilibrium is stable. Since in Lemma 4.4 we have established a priori bounds for the roots, we know that roots can enter the right half-plane only through a compact subset of the imaginary axis. Now note that the signs of denominator in (5.2) and numerator in (5.3) agree, and the signs of the denominator of (5.3) and the determinant of Lemma 5.3 are opposite. It follows that, as long as the curves in the (*m*, *r*)-plane are in the positive quadrant, the sign of the determinant is negative. Hence we can combine the previous arguments with considerations on the ordering of the curves, defined on the intervals bounded by the singularities, to conclude that the boundary between the maximal region of stability and the maximal region of instability is given by the curve $\omega \mapsto (m,r)(\omega ) \in {\mathbb {R}}_+^2$, with

5.4 $$\begin{aligned} \omega \in {\left\{ \begin{array}{ll} (\omega _1,\omega _2),&{}\mathrm{if}\;\eta <1\\ (0,\omega _1),&{}\mathrm{if}\;\eta \ge 1. \end{array}\right. } \end{aligned}$$

**Fig. 4:**
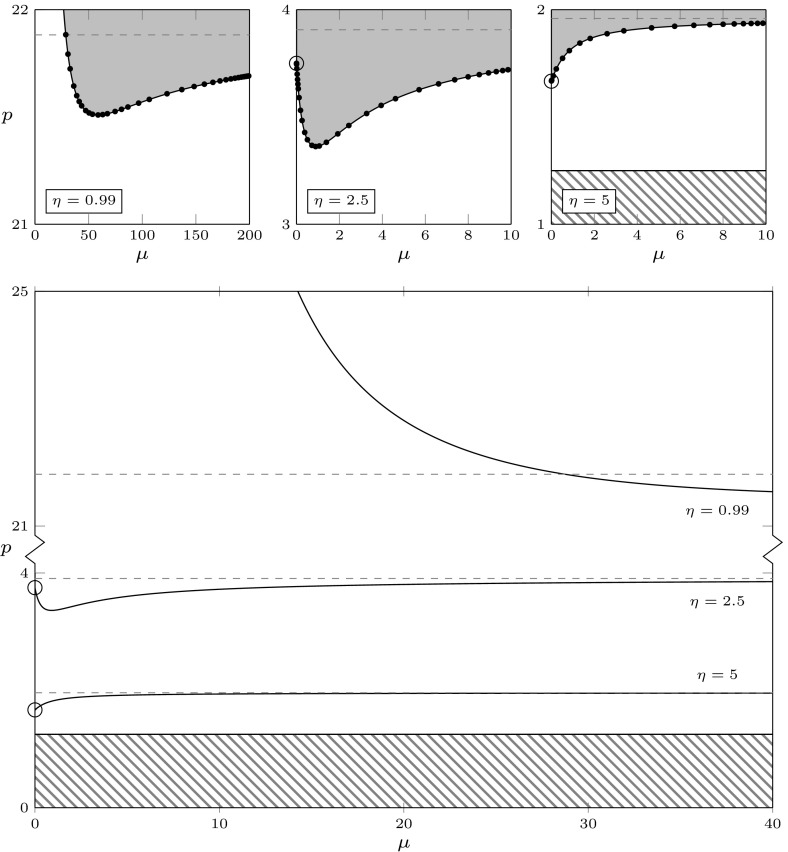
Existence and stability of equilibria in the plane $(\mu ,p)$, for the rate specifications of Lemma 4.5(e), with $\mu _w=1$ and $a=0.9$. The positive equilibrium exists for $p>\mu _w/(2a-1)=1.25$ (no positive equilibrium in the striped region). In the large panel, the solid curves are the analytical curves (5.2) and (5.3) and show how the stability boundary changes qualitatively with $\eta$: the positive equilibrium is stable below the curves, unstable above. The three upper panels contain some zooms of the curves: the instability region is shaded and the black dots are the numerical approximations of the curves computed by numerical continuation with the software dde-biftool. The rates correspond to the specifications (s)$_\text {d}$ and (pv)$_0$ in Table 2

See Fig. 4 for a plot. We refer to Chapter XI of Diekmann et al. (1995) for further details on the argumentation above.

### Analysis of the stability boundary

In the following, we use $f(\omega ^+)$ and $f(\omega ^-)$ as a short-hand notation for the one-sided right and left limit of a function *f*, respectively, whenever they exist, i.e.,

$$\begin{aligned} f(\omega ^+) = \lim _{\omega \rightarrow \omega ^+} f(\omega ),\qquad f(\omega ^-) = \lim _{\omega \rightarrow \omega ^-} f(\omega ). \end{aligned}$$

From Lemma 5.2 it becomes clear that, if $\eta <1$,

$$\begin{aligned}&(m,r)(\omega _1^-)=(0,-\infty ),&(m,r)(\omega _2^-)=(+\infty ,r(\omega _2)), \\&(m,r)(\omega _1^+)=(0,+\infty ),&(m,r)(\omega _2^+)=(+\infty ,r(\omega _2)), \end{aligned}$$

and, if $$\eta >1$$,

$$\begin{aligned}&(m,r)(0)=(0,r(0))=\left( 0,\frac{1}{\eta -1}\right) , \; \text { and }\\&(m,r)(\omega _1^-)=(+\infty ,r(\omega _1)), \quad (m,r)(\omega _1^+)=(-\infty ,r(\omega _1)), \end{aligned}$$

with $r(\omega _1),r(\omega _2)\in (0,\infty )$, see Fig. 3. Note that, for $\eta \rightarrow 1^+$, $r(0)\rightarrow + \infty$. The stability region is unbounded in *m*, whereas it is bounded in *r* if $\eta \ge 1$, and unbounded if $\eta <1$. In the following we present a rather complete analysis of the gradient of the stability boundary. We refer to Fig. 4 for a visual summary.

#### Lemma 5.4

One has $m'(\omega )>0$ on $(0,\omega _1)$ for $\eta \ge 1$, and on $(\omega _1,\omega _2)$ for $\eta <1$.

It follows that we can alternatively define the stability boundary via (5.2) and (5.3) as $m\mapsto r(m)\in {\mathbb {R}}_+$, with

$$\begin{aligned} m \in \left\{ \begin{array}{ll} (0,\infty ),&{}\quad \text { if } \eta <1\\ {[}0,\infty ),&{}\quad \text { if } \eta \ge 1. \end{array}\right. \end{aligned}$$

#### Lemma 5.5

For $\eta <3$ the stability boundary $m\mapsto r(m)$ first decreases, then increases. Here, the minimum is assumed in values $\omega >\frac{\pi }{2}$ if $\eta <\frac{\pi ^2}{4}$, $\omega =\frac{\pi }{2}$ if $\eta =\frac{\pi ^2}{4}$, and $\omega <\frac{\pi }{2}$ if $\eta >\frac{\pi ^2}{4}$. For $\eta \ge 3$ the curve is increasing.

Note that for $\eta >1$ there is a turning point after the minimum. Moreover, for both cases $\eta <1$ and $\eta \ge 1$, the proofs of the previous results show (although Fig. 4 does not) that $r'(0)=0$ and that there is a turning point also before the minimum.

## Transformation to fixed delay for *g* independent of maturity

In this section we restrict to the case of a maturation rate *g* independent of maturity (i.e., $D_1g\equiv 0$), and we establish a relation between the solutions of the SD-DDE and the solutions of a special equation with fixed delay. When $g(x,v)=g(v)$, Eq. (2.8) reads

6.1 $$\begin{aligned} v'(t) = \frac{\gamma (v(t-\tau (v_t))) g(v(t)) w(t-\tau (v_t))}{g(v(t-\tau (v_t)))}\mathop {}\!\mathrm {e}^{\int _0^{\tau (v_t)} d(y(s,v_t),v(t-s))\mathop {}\!\mathrm {d}s} - \mu v(t).\nonumber \\ \end{aligned}$$

In this case, the explicit expression

6.2 $$\begin{aligned} y(s,\psi ) = x_2-\int _{-s}^0 g(\psi (\theta ))\mathop {}\!\mathrm {d}\theta , \quad s\in [0,h], \end{aligned}$$

allows us to write Eq. (2.6) as

6.3 $$\begin{aligned} \int _{-\tau }^{0} g(\psi (\theta ))\mathop {}\!\mathrm {d}\theta = \delta , \end{aligned}$$

with

6.4 $$\begin{aligned} \delta :=x_2-x_1. \end{aligned}$$

Next to (2.7) and (6.1), we consider the differential equations

6.5 $$\begin{aligned} \omega '(\phi )&= \frac{q(u(\phi ))}{g(u(\phi ))} \omega (\phi ) \end{aligned}$$

6.6 $$\begin{aligned} u'(\phi )&= \frac{\gamma (u(\phi -\delta )) \omega (\phi -\delta )}{g(u(\phi -\delta ))} \mathop {}\!\mathrm {e}^{\int _0^{\delta } \frac{d(x_2-s,u(\phi -s))}{g(u(\phi -s))}\mathop {}\!\mathrm {d}s} - \frac{\mu u(\phi )}{g(u(\phi ))}, \end{aligned}$$

for $\phi > 0$, where the maximum delay $\delta$ is fixed by the model parameters, see (6.4). The system (6.5) and (6.6) is provided with the initial condition

6.7 $$\begin{aligned} (\omega ,u)_0=(\eta ,\zeta ), \end{aligned}$$

for $(\eta ,\zeta )$ belonging to the solution manifold. We will explicitly construct a transformation of the independent variable that, given a solution of (2.7) and (6.1), provides a solution of (6.5) and (6.6), and vice versa.

Given an interval $J\subseteq {\mathbb {R}}$ with $0\in J$ and a continuously differentiable function *f* defined on *J* with range in the domain of *g*, we define the transformation $\varPhi _f :J \rightarrow {\mathbb {R}}$ such that

$$\begin{aligned} \varPhi _f(t) := {\left\{ \begin{array}{ll} \int _0^t g(f(\theta ))\mathop {}\!\mathrm {d}\theta , &{} \text {if } t\ge 0,\\ -\int _t^0 g(f(\theta ))\mathop {}\!\mathrm {d}\theta , &{} \text {if } t<0.\\ \end{array}\right. } \end{aligned}$$

Note that $\varPhi _f(0)=0$ and $\varPhi _f$ is $C^1$ with $\varPhi _f'(t)=g(f(t))$ for $t \in J$.

Similarly, we define the transformation $T_f :J \rightarrow {\mathbb {R}}$ such that

$$\begin{aligned} T_f(\phi ) := {\left\{ \begin{array}{ll} \int _0^{\phi } \frac{1}{g(f(\theta ))}\mathop {}\!\mathrm {d}\theta , &{}\text {if } \phi \ge 0, \\ -\int _{\phi }^0 \frac{1}{g(f(\theta ))}\mathop {}\!\mathrm {d}\theta , &{}\text {if } \phi <0. \end{array}\right. } \end{aligned}$$

Note that $T_f(0)=0$ and $T_f$ is $C^1$ with $T_f'(\phi )=1/g(f(\phi ))$ for $\phi \in J$.

### Lemma 6.1

Let *v* be defined in $[-h,\infty )$ and *u* be defined in $[-\delta ,\infty )$, both with range in the domain of *g*. Define $\zeta :=u_0$ and $\tau _{\zeta } := -T_{\zeta }(-\delta )$. Then $\varPhi _v$ and $T_u$ are invertible with $\varPhi _v^{-1}\in C^1([-\delta ,\infty ),{\mathbb {R}})$ and $T_u^{-1} \in C^1([-\tau _{\zeta },\infty ),{\mathbb {R}})$.

We are now ready to specify the relation between the solutions of the initial value problems.

### Theorem 6.2

Let (*w*, *v*) be a solution of (2.7) and (6.1) with initial condition (3.6) defined on $[-h,\infty )$, and let $\eta = \varphi \circ \varPhi _{\psi }^{-1}$, $\zeta =\psi \circ \varPhi _{\psi }^{-1}$. Then $(\omega ,u)$ defined by

6.8 $$\begin{aligned} \omega := w\circ \varPhi _v^{-1},\quad u:= v\circ \varPhi _v^{-1}, \end{aligned}$$

is a solution of (6.5)–(6.7) on $[-\delta ,\infty )$.

Vice versa, given a solution $(\omega ,u)$ of (6.5)–(6.7) on $[-\delta ,\infty )$, define $\tau _{\zeta }:=-T_{\zeta }(-\delta )$, and let $\varphi ,\psi$ defined on $[-h,0]$ such that

6.9 $$\begin{aligned} \varphi \big |_{[-\tau _{\zeta },0]} = \eta \circ T_{\zeta }^{-1} \quad \text { and } \quad \psi \big |_{[-\tau _{\zeta },0]} = \zeta \circ T_{\zeta }^{-1}. \end{aligned}$$

Then, (*w*, *v*) defined on $[-\tau _{\zeta },\infty )$ by

6.10 $$\begin{aligned} w:= \omega \circ T_u^{-1},\quad v:= u\circ T_u^{-1}, \end{aligned}$$

and extended to $[-h,-\tau _{\zeta })$ such that $w_0=\varphi$, $v_0=\psi$, is a solution of (2.7) and (6.1).

We stress that, while the solution $(\omega ,u)$ of the initial value problem (6.5)–(6.7) is uniquely defined from (*w*, *v*), the converse is not true: the construction of a solution (*w*, *v*) defined on $[-h,\infty )$ from a given solution $(\omega ,u)$ in general involves an arbitrary extension on $[-h,-\tau _{\zeta })$. Such extension is required in the mathematical analysis to define the state of the dynamical system on the interval $[-h,0]$. However, this should not cause any problem in applications, where usually the initial data $(\varphi ,\psi )$ for system (2.7) and (6.1) is fixed by observations or chosen arbitrarily for experiments. From a biological point of view, the transformation $\phi =\varPhi _v(t)$ translates the temporal time-scale into the “physiological” time-scale of progenitor cells (i.e., we measure the maturity level of cells instead of their age).

We consider the transformed functional *G*, which is given as

$$\begin{aligned} G_1(\varphi ,\psi )&=\frac{q(\psi (0))}{g(\psi (0))}\varphi (0) \\ G_2(\varphi ,\psi )&=\frac{\gamma (\psi (-\delta ))\varphi (-\delta )}{g(\psi (-\delta ))}\mathop {}\!\mathrm {e}^{\int _0^\delta \frac{d(x_2-s,\psi (-s))}{g(\psi (-s))}\mathop {}\!\mathrm {d}s} -\frac{\mu \psi (0)}{g(\psi (0))}. \end{aligned}$$

Note that $F(w,v)=0$ if and only if $G(w,v)=0$. We now let (*w*, *v*) denote the positive equilibrium. We remark that results similar to the following hold for the trivial equilibrium. We will not go into details.

### Proposition 6.3

For $k=1/g(v)$ it holds that $\det (z-DF(w,v)e_z)=0$ if and only if $\det (kz-DG(w,v)e_{kz})=0$.

The result is a simple corollary of the following

### Lemma 6.4

For $k=1/g(v)$ and $z\in {\mathbb {C}}$ we have

$$\begin{aligned} DG(w,v)e_{kz}=kDF(w,v)e_z. \end{aligned}$$

Therefore, system (2.7) and (6.1) and system (6.5)–(6.6) have the same equilibria, with the same stability properties.

## Numerical stability analysis: the pseudospectral approach for differential equations with state-dependent delay

In this section we adapt the pseudospectral discretisation approach, presented by Breda et al. (2016a) in the case of nonlinear delay equations with fixed delay, to the case when the delay depends on the state. The main idea of spectral methods is to project a given equation defined on an infinite-dimensional space into a subspace of finite dimension, see Gottlieb and Orszag (1977). Pseudospectral techniques are particular kinds of spectral methods where the approximating space is chosen as the space of polynomials of a fixed degree $M\in {\mathbb {N}}$, and the projection is done through *collocation*: a finite number of conditions is imposed on a set of points, called *collocation nodes*.

Breda et al. (2016a) showed that, by means of pseudospectral techniques, it is possible to obtain a finite-dimensional system of ordinary differential equations that approximates the dynamical properties of the original delay equation with fixed delay. Here, we extend the method to equations where the delay is state-dependent by exploiting the uniform bound of the delay (3.5). Thanks to the latter, the pseudospectral approach can be applied on the fixed interval $[-h,0]$, and the convergence analysis that holds in the fixed-delay case remains true also in state-dependent case, as we will explain below. For the sake of presentation, we summarise the main steps of the method and write explicitly the approximating system of ODE for Eqs. (2.7) and (2.8). We refer to Breda et al. (2016a) for further details and for more general types of delay equations.

The existence and uniqueness of solutions of (2.7)–(2.8) with initial condition (3.6) implies that the semigroup of solution operators is well defined, strongly continuous, and its infinitesimal generator is

$$\begin{aligned} {\mathcal {A}}(\varphi ,\psi ) = (\varphi ',\psi '), \quad (\varphi ,\psi )\in X, \end{aligned}$$

where *X* is defined in (3.4), see Crandall and Liggett (1971). Note that the action of ${\mathcal {A}}$ is linear, while its domain *X* is defined via a nonlinear condition. Then, (2.7)–(2.8) with initial condition (3.6) is equivalent to the abstract differential equation

7.1 $$\begin{aligned} \frac{\mathop {}\!\mathrm {d}}{\mathop {}\!\mathrm {d}t}({\mathcal {W}}(t),{\mathcal {V}}(t)) = {\mathcal {A}}({\mathcal {W}}(t),{\mathcal {V}}(t)), \quad t\ge 0, \end{aligned}$$

for $({\mathcal {W}}(t),{\mathcal {V}}(t)) \in X$, with initial condition

7.2 $$\begin{aligned} ({\mathcal {W}}(0),{\mathcal {V}}(0))=(\varphi ,\psi ). \end{aligned}$$

The problems are equivalent in the sense that, if (*w*, *v*) is a solution of (2.7)–(2.8) and (3.6), then $({\mathcal {W}},{\mathcal {V}})$ is a solution of (7.1)–(7.2) with ${\mathcal {W}}(t)=w_t$, ${\mathcal {V}}(t)=v_t$ for all $t\ge 0$, and vice versa.

Equation (7.1) is a differential equation in the space of continuous functions. In order to obtain a numerical approximation, we fix a positive integer $M\in {\mathbb {N}}$, called *discretisation index*, and define $\varPi _M$ as the space of ${\mathbb {R}}$-valued polynomials of degree *M*. Then we consider the finite-dimensional space $X_M := \varPi _M\times \varPi _M$. The projection of the abstract differential Eq. (7.1) in the space $X_M$ is

7.3 $$\begin{aligned} \frac{\mathop {}\!\mathrm {d}}{\mathop {}\!\mathrm {d}t}({\mathcal {W}}_M(t),{\mathcal {V}}_M(t)) = {\mathcal {A}}_M({\mathcal {W}}_M(t),{\mathcal {V}}_M(t)), \quad t\ge 0, \end{aligned}$$

where $({\mathcal {W}}_M(t),{\mathcal {V}}_M(t))\in X_M$, and $\mathcal A_M :X_M \rightarrow X_M$ is called the approximating generator. The operator ${\mathcal {A}}_M$ is defined by imposing some collocation conditions on a finite set of nodes, as explained for instance by Gottlieb and Orszag (1977) and Boyd (2001), in the following way. We introduce a mesh of collocation nodes, $\varOmega _M:=\{\theta _0,\dots ,\theta _M\} \subset [-h,0]$, such that

$$\begin{aligned} -\,h = \theta _M< \theta _{M-1}< \cdots < \theta _0=0. \end{aligned}$$

Given $M+1$ values $z_0,\dots ,z_M$, their interpolating polynomial on $\varOmega _M$ is uniquely determined, and it can be represented in Lagrange form as a linear combination of the values $z_j$ as

7.4 $$\begin{aligned} p_M(\theta ;z_0,\dots ,z_M) := \sum _{j=0}^M \ell _j(\theta ) z_j, \quad \theta \in [-h,0], \end{aligned}$$

where the coefficients

$$\begin{aligned} \ell _j(\theta ):= \prod _{k\ne j} \frac{\theta - \theta _k}{\theta _j-\theta _k}, \quad j=0,1,\dots ,M, \end{aligned}$$

form the basis of Lagrange polynomials, as explained for instance by Quarteroni et al. (2007) in Chapter 8. Note that $\ell _j(\theta _k)=\delta _{jk}$, where $\delta _{jk}$ is the Kronecker’s symbol. Note also that, by linearity, the derivative of $p_M$ at the nodes can be expressed as

7.5 $$\begin{aligned} \frac{\mathop {}\!\mathrm {d}p_M}{\mathop {}\!\mathrm {d}\theta } (\theta _k;z_0,\dots ,z_M) = \sum _{j=0}^M \ell _j'(\theta _k) z_j = \sum _{j=0}^M d_{kj} z_j, \quad k=0,1,\dots ,M, \end{aligned}$$

where

$$\begin{aligned} d_{kj} := \ell _j'(\theta _k), \quad j,k=0,1,\dots M, \end{aligned}$$

are the elements of the differentiation matrix corresponding to the Lagrange polynomial basis. Note that the elements $d_{kj}$ can be explicitly computed by established numerical routines, as explained by Trefethen (2000).

Following Breda et al. (2016a), for all $({\mathcal {W}}_M,{\mathcal {V}}_M)\in X_M$ we define ${\mathcal {A}}_M({\mathcal {W}}_M,{\mathcal {V}}_M)$ as the pair of polynomials satisfying

$$\begin{aligned} {\mathcal {A}}_M({\mathcal {W}}_M,{\mathcal {V}}_M)(\theta _j)&:= {\left\{ \begin{array}{ll} F({\mathcal {W}}_M,{\mathcal {V}}_M) &{} \text {if } j=0 \\ ({\mathcal {W}}_M',{\mathcal {V}}_M')(\theta _j), &{}\text {if } j=1,\dots ,M, \end{array}\right. } \end{aligned}$$

where $F=(F_1,F_2)$ is the functional defining the right-hand side of the DDE, as defined at the beginning of Sect. 3. Note that ${\mathcal {A}}_M$ is well defined because every *M*-degree polynomial is uniquely determined by $M+1$ independent conditions. By exploiting the representations (7.4) and (7.5) into (7.3), it is easy to obtain the following result.

### Proposition 7.1

The pseudospectral approximation of system (2.7)–(2.8) reads

7.6 $$\begin{aligned} \left\{ \begin{array}{l} w_0'(t) = F_1({\mathcal {W}}_M,{\mathcal {V}}_M)\\ v_0'(t) = F_2({\mathcal {W}}_M,{\mathcal {V}}_M)\\ w_1'(t) = d_{10} w_0(t) + d_{11} w_1(t) + \cdots + d_{1M} w_M(t)\\ v_1'(t) = d_{10} v_0(t) + d_{11} v_1(t) + \cdots + d_{1M} v_M(t)\\ \vdots \\ w_M'(t) = d_{M0} w_0(t) + d_{M1} w_1(t) + \cdots + d_{MM} w_M(t)\\ v_M'(t) = d_{M0} v_0(t) + d_{M1} v_1(t) + \cdots + d_{MM} v_M(t), \end{array} \right. \end{aligned}$$

for $$w_j(t),v_j(t)\in {\mathbb {R}}$$, $$j=0,\dots ,M$$, where

$$\begin{aligned} {\mathcal {W}}_M := p_M(\cdot ;w_0(t),\dots ,w_M(t)), \quad {\mathcal {V}}_M := p_M(\cdot ;v_0(t),\dots ,v_M(t)). \end{aligned}$$

The polynomials ${\mathcal {W}}_M$ and ${\mathcal {V}}_M$ are the pseudospectral projections of *w*(*t*) and *v*(*t*), respectively.

The system (7.6) consists of $2(M+1)$ ODE, and its properties can be studied with well-established software like matcont for matlab.

In practice, the interpolation nodes are chosen as

$$\begin{aligned} \theta _{j}=\frac{h}{2}\left( \cos \left( \frac{j\pi }{M}\right) -1\right) , \quad j=0,1,\ldots ,M. \end{aligned}$$

In the literature, these points are known as Chebyshev extremal nodes, and they are defined as the abscissas corresponding to the maxima and minima of Chebyshev orthogonal polynomials. Chebyshev nodes ensure that the interpolation process converges on functions that are at least Lipschitz continuous, as explained for instance by Davis (1975) and Quarteroni et al. (2007). Furthermore, the interpolation of infinitely differentiable functions converges with *spectral accuracy*, viz. the error decays faster than $O(M^{-k})$ for any integer *k*, as shown by Trefethen (2000, 2013).

The one-to-one correspondence of the equilibria of (2.7)–(2.8) and (7.6) can be proven in the same way as done by Breda et al. (2016a). Moreover, the fact that the states of the dynamical system are defined in $[-h,0]$ allows us to use convergence results about interpolation on a bounded and fixed interval. Since the eigenfunctions associated with the linearised system are exponential, we can exploit the spectral convergence of the interpolation process and use the argument of Breda et al. (2005) to ensure the convergence of the associated characteristic roots. In particular, the eigenvalues corresponding to the linearisation of (7.6) approximate the rightmost eigenvalues of the linearisation of (7.1) with spectral accuracy, as proven by Breda et al. (2005, 2015a, b). In conclusion, from Theorem 2.4 and Corollary 2.7 of Breda et al. (2016a), by using results on interpolation on $[-h,0]$, the following result follows.

### Proposition 7.2

If (*w*, *v*) is an equilibrium of (2.7)–(2.8), then

7.7 $$\begin{aligned} (w_0,v_0,\dots ,w_M,v_M) \in {\mathbb {R}}^{2(M+1)} \end{aligned}$$

with

7.8 $$\begin{aligned} w_j=w, \quad v_j=v,\quad j=0,\dots ,M, \end{aligned}$$

is an equilibrium of (7.6). Vice versa, if (7.7) is an equilibrium of (7.6), then (7.8) holds and (*w*, *v*) is an equilibrium of (2.7–2.8). Moreover, the characteristic roots and the bifurcation points associated with the equilibria of (7.6) converge to those of the corresponding equilibria of (2.7)–(2.8) with spectral accuracy when *M* increases.

**Fig. 5:**
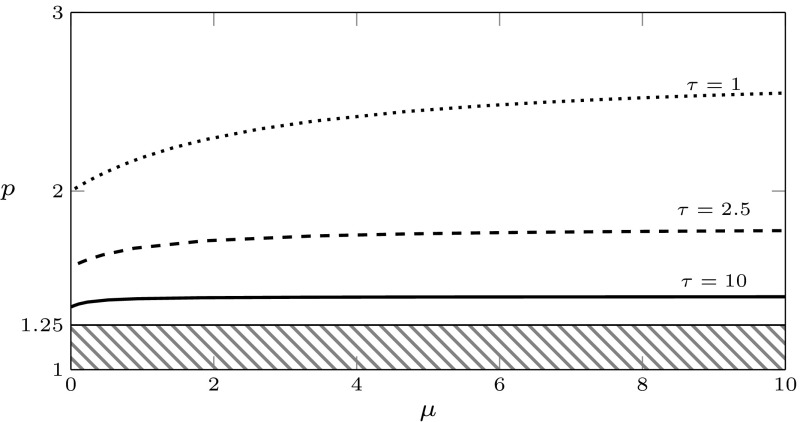
Stability boundary for parameter set (pv)$_0$ and (s)$_{\text {s}}$, for different values of $\tau$. The positive equilibrium exists for $p>1.25$, and it is stable in the region below the stability boundary

**Fig. 6:**
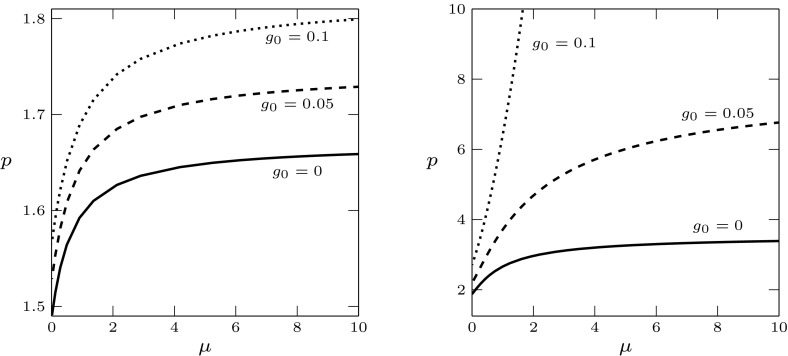
Stability boundary for parameter set (pv)$_1$ and different values of $g_0$. Left: stem cell regulation (s)$_\text {s}$; right: (s)$_\text {d}$. Stability region below the boundary

**Fig. 7:**
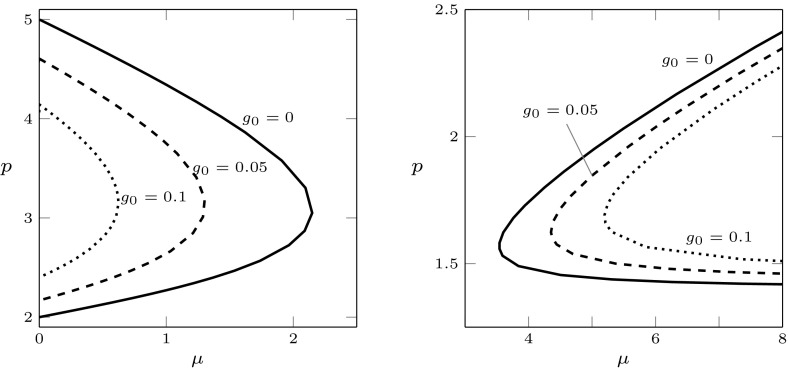
Stability boundary for parameter set (pv)$_2$ and different values of $g_0$. Left: stem cell regulation (s)$_\text {s}$, stability region to the right of the boundary; right: (s)$_\text {d}$, stability region to the left of the boundary

In the next section, we first validate the pseudospectral approximation technique on some instances of system (2.7)–(2.8) that can be transformed via the time transformation introduced in Sect. 6. In particular, the numerical output of the pseudospectral approximation (for $M=15$ and tolerance $10^{-6}$) was compared with the output of dde-biftool on the fixed-delay reformulation. All the curves in Figs. 4, 5, 6 and 7 were computed both with dde-biftool and with the pseudospectral method, and all the respective curves are indistinguishable to the eye. Since dde-biftool is a well-established package for numerical bifurcation of delay equations, we take the agreement of the numerical output as an important evidence of the validity of the pseudospectral discretisation approach applied to a type of equations (viz., SD-DDE) to which it has never been applied before. After the validation of the method, we exploit the pseudospectral approach to approximate the stability boundary of system (2.7) and (2.8) in some cases when the time transformation is not applicable and there is no well-established software available for bifurcation analysis.

## Numerically computed stability boundaries

For the numerical results in this section we consider the stem cell rates specified in Table 1 and we fix the following parameters

8.1 $$\begin{aligned} x_1=0,\quad x_2=1,\quad \mu _w=1, \quad a=0.9. \end{aligned}$$

For this choice of parameters, condition (4.10) for the existence of a positive equilibrium (*w*, *v*) reduces to $p>1.25$. As proven in Sect. 4, the positive equilibrium is stable upon emergence. We consider two different regulation mechanisms of stem cell processes (cfr. also Tables 1 and 2):

$$\hbox {(s)}_\text {s}$$
regulated self-renewal, unregulated division: $k_a=1$, $k_p=0$,
$$\hbox {(s)}_\text {d}$$
unregulated self-renewal, regulated division: $k_a=0$, $k_p=1$.

In the following we investigate numerically the stability region of the positive equilibrium by including different types of *v*- and *x*-dependence in the maturation rate of progenitor cells.

**Table 2:** Summary of the parameter sets used in the numerical tests

	Parameters	Description
(s)$_\text {s}$	$k_a=1$, $k_p=0$	Stem cell regulated self-renewal, unregulated division (see Table 1)
(s)$_\text {d}$	$k_a=0$, $k_p=1$	Stem cell unregulated self-renewal, regulated division (see Table 1)
(pv)$_0$	$k_1=0$, $k_2=0$	Progenitor maturation independent of *v* (see (8.2))
(pv)$_1$	$k_1=1$, $k_2=0$	Progenitor maturation decreasing in *v* (see (8.2))
(pv)$_2$	$k_1=0$, $k_2=1$	Progenitor maturation increasing in *v* (see (8.2))
(px)$_1$	$p_u(x)= 0.3$	First type of progenitor *x*-dependence (see (8.2) and Table 3)
(px)$_2$	$p_u(x)= p$	Second type of progenitor *x*-dependence (see (8.2) and Table 3)

See also Table 1 for specifications of stem cell rates, and Eq. (8.2) and Table 3 for the progenitor maturation rate

**Table 3:** Specification of *x*-dependent rates for progenitor cells, see also Fig. 8

	$p_u(x)$	$a_u(x)$	
(px)$_1$	$p_u(x)= 0.3$	Constant:	$a_u(x)=1/3$
		Linear:	$a_u(x)=2/3(1-x)$
		Quadratic:	$a_u(x)=0.1+2.8(x-0.5)^2$
(px)$_2$	$p_u(x)= p$	Constant:	$a_u(x)=0.9$
		Linear:	$a_u(x)=0.99-0.18x$
		Quadratic:	$a_u(x)=0.855-0.54(x-0.5)^2$

In the modelling literature we could find very few specifications of how the maturation speed depends on maturity. Exceptions are the maturation rate proposed by Doumic et al. (2011), which is increasing, and by Alarcón et al. (2011), which is decreasing. Here we choose a maturation rate that allows to incorporate both cases. Hence, we specify the maturation rate of the form

8.2 $$\begin{aligned} g(x,v)= g_0 + 2 \frac{p_u(x)}{1+k_1v} \left( 1- \frac{a_u(x)}{1+k_2v}\right) . \end{aligned}$$

By specifying the coefficients $a_u(x)$ and $p_u(x)$ we can vary the dependence on *x*; for illustration, we will consider two types of dependence that are mathematically convenient, see Table 3. We note that some parameter sets for the division rate and the fraction of self-renewal of cells in progenitor compartments are proposed by Stiehl et al. (2014).

In the following we also assume that the net production of progenitor cells is zero, i.e., $d(x,v)\equiv 0$, corresponding to a biological situation when progenitor death and self-renewal are in balance.

### Constant progenitor maturation rate

We first consider the case of constant maturation rate of progenitor cells by taking $k_1=k_2=0$ (case (pv)$_0$ in Table 2), and constant $p_u(x)$ and $a_u(x)$, so that $g(x,v)=g$. This means that the maturation process is not regulated by mature cells, and the maturation speed is the same at all maturity levels. In this case, (2.7–2.8) reduces to an equation with one fixed discrete delay $\tau =\delta /g$, whose bifurcation properties can be numerically analysed with the package dde-biftool.

The case (s)$_\text {d}$ corresponds to the specifications of Lemma 4.5(e). The stability boundary was studied analytically in Sect. 5. The upper panels in Fig. 4 show the analytical stability boundary (solid line) and the numerically computed stability boundary obtained with dde-biftool (black dots) for different values of $\tau$ (we recall that $\eta =\tau$ for the choice (8.1)).

In the case (s)$_\text {s}$, we studied the stability boundary for different values of $\tau$ varying between 0.1 and 50. Some examples are plotted in Fig. 5. The stability boundary moves towards higher values of *p* when the delay $\tau$ decreases. In this case the numerical simulations did not show any minimum: this behaviour is different from the case (s)$_\text {d}$, where a minimum appears for small values of $\tau$, see Fig. 4.

### Maturation rate dependent on the amount of mature cells

We assume now that the maturation rate of a progenitor cell is regulated by the amount *v* of mature cells, but does not depend on the maturity of the cell itself: $g(x,v)=g(v)$. Under these assumptions, we can apply the time transformation introduced in Sect. 6, so that the equilibria of system (2.7) and (2.8) and their stability properties are the same as in the fixed-delay system (6.5)–(6.6). We investigate numerically the latter system with dde-biftool. In particular, we take (8.2) with

$$\begin{aligned} a_u(x)=a,\quad p_u(x)=p, \end{aligned}$$

and we consider two cases (see also Table 2):

$$\hbox {(pv)}_1$$
maturation rate decreasing with *v*: $k_1=1$, $k_2=0$,
$$\hbox {(pv)}_2$$
maturation rate increasing with *v*: $k_1=0$, $k_2=1$.

In the case (pv)$_1$, the numerically computed stability boundary does not show a minimum for the parameter values considered in the simulations. Some examples are plotted in Fig. 6. When increasing $g_0$, the boundary moves up. Interestingly, under assumption (s)$_\text {d}$ we observe a change in the concavity of the boundary when $g_0$ is large enough, see Fig. 6 (right). As evident from the figure, by changing from concave to convex, the stability region of the equilibrium becomes much larger and perhaps unbounded in *p*, promoting stability of the system at equilibrium.

**Fig. 8:**
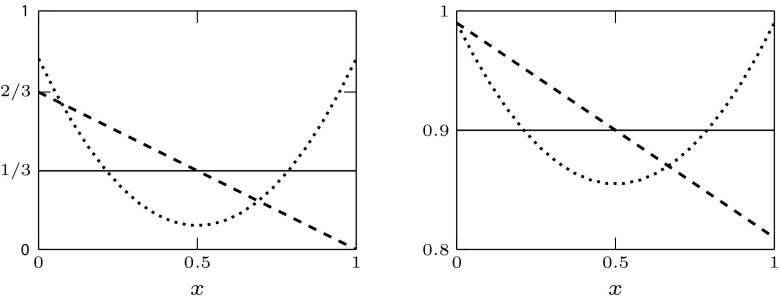
Plot of $a_u(x)$ from Table 3: linear (dashed), quadratic (dotted) and constant mean value (solid). Left: (px)$_1$; right: (px)$_2$

The type of regulation (pv)$_2$ is elaborated by Doumic et al. (2011). In this case, the numerical results reveal that the stability region of the positive equilibrium has a qualitatively different shape compared to the cases examined so far, see Fig. 7. In both cases (s)$_\text {s}$ and (s)$_\text {d}$, the plots suggest the existence of an interval of values of $\mu$ such that the positive equilibrium is stable for every value of *p*, but in the first case this happens for large values of $\mu$, while in the second case this happens for small values of $\mu$. This motivated us to investigate also the case of simultaneous stem cell regulation ($k_a=k_p=1$). No destabilisation was detected when varying the $\mu$ and *p* in the interval (0, 50). Thus, the regulation of the stem cell processes seem to have a significant impact on the shape of the stability region when the maturation rate is regulated by amount of mature cells only.

**Fig. 9:**
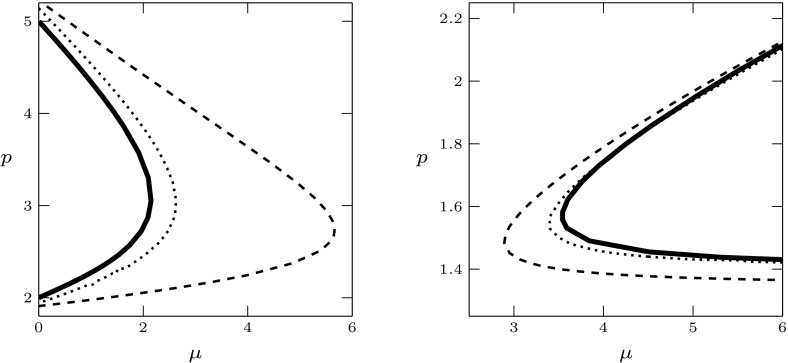
Stability boundary for case (px)$_2$ in Table 3, when $a_u(x)$ is constant (solid), linear (dashed) and quadratic (dotted), and *v*-dependence (pc)$_2$. Left: stem cell regulation (s)$_\text {s}$, stability region to the right of the boundary; right: (s)$_\text {d}$, stability region to the left of the boundary

**Fig. 10:**
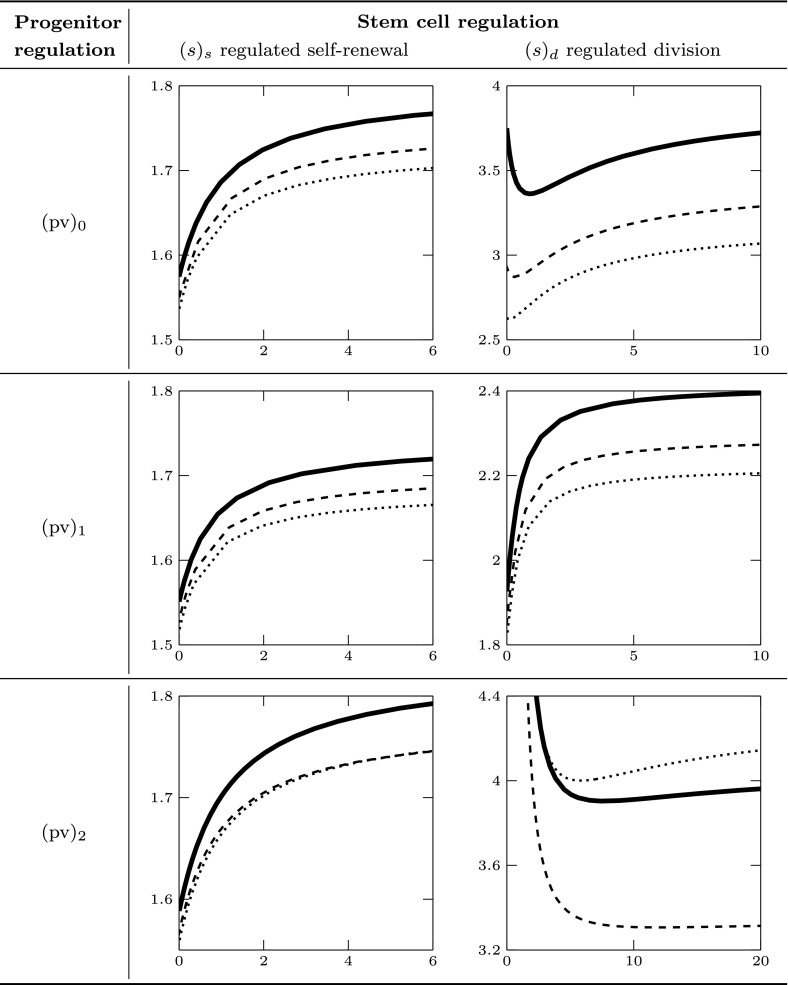
Stability boundary in the plane $(\mu ,p)$ for case (px)$_1$ in Table 3, when $a_u(x)$ is constant (solid), linear (dashed) and quadratic (dotted). Different rows correspond to different types of *v*-dependence, see also Table 2. Stability region is below the boundary

### Maturation rate dependent on maturity and on the amount of mature cells

Finally, we investigate the case when the maturation rate depends explicitly on maturity *x*. We consider (8.2) with $g_0=0$ and maturity-dependent rates specified in Table 3. As clear from the specifications, we fix $p_u$ constant and we compare two different forms of the function $a_u(x)$ (linear decreasing and quadratic) with their mean constant value, see also Fig. 8. A linear increasing function $a_u(x)$ did not produce qualitatively different results, so we did not include the analysis here. Moreover, an increasing $a_u(x)$ is not supported by the parameter sets proposed by Stiehl et al. (2014). We stress again that the transformation introduced in Sect. 6 does not apply to this case, and there is no well-established software for the numerical bifurcation analysis of (2.7)–(2.8). Therefore we study the system numerically by applying the pseudospectral discretisation method described in Sect. 7 and by studying the approximating system (7.3) with the numerical package matcont. In the following simulations, the discretisation index *M* is chosen as an integer in the interval [10, 15] that guarantees the convergence of the computation.

With parameter set (px)$_1$, we considered different types of *v*-dependence corresponding to the parameter sets (pv)$_0$, (pv)$_1$ and (pv)$_2$ summarised in Table 2. The results are summarised in Fig. 10. For parameter set (px)$_2$, for illustration we only considered the case (pv)$_2$ of maturation rate increasing with *v* (the case (pv)$_1$ did not exhibit any more interesting behaviour). The output is plotted in Fig. 9.

Introducing the dependence on maturity did not produce any qualitative change in the stability boundary compared to Figs. 5, 6 and 7, but affected the location of the boundary, sometimes in a relevant way, see for instance Fig. 9 (left). It is interesting to notice that the quadratic *x*-dependence in Fig. 10, (pv)$_2$ (right), has a different effect (stability boundary moves up) compared to the other figures, where the stability boundary moves down. Also, in Fig. 9 we observe that the boundary associated with the quadratic dependence is located between those associated with linear and constant rates, differently from the outcomes of Fig. 10. These differences in the stability boundaries lead us to believe that the effects of maturity dependence are of great complexity and strongly dependent on the specifications of model parameters.

## Discussion and outlook

In the present paper we have considered a model for the regulated maturation process of stem cells. We have developed analytical and numerical methods and used these to show that a stable positive equilibrium destabilises the trivial in a transcritical bifurcation, and that, upon further variation of parameters, the positive equilibrium may destabilise. Note that we could introduce a quantity *R* that can be interpreted as the expected lifetime net offspring production of a stem cell in absence of regulation. In the special case analysed in Sect. 5 (with the rates specified in Lemma 4.5(e), i.e., fixed delay only), this quantity would be $R:=(2a-1)p/\mu _w$ and, by considering *R* as bifurcation parameter, the transcritical bifurcation occurs at $R=1$ as in classical population ecology and epidemiology, see for instance Diekmann et al. (1990). We note that, in the case of regular cell production, the healthy state at homeostasis corresponds to the positive equilibrium. If the model describes cancer stem cells, the positive equilibrium may represent a pathological situation, while the zero equilibrium may correspond to the absence of cancer tissue.

One motivation for the present research is the analysis of a six-compartment model of Marciniak-Czochra et al. (2009). By means of simulations the authors show that, under various choices of regulation mode and parameters, total cell numbers converge to a positive equilibrium, which is associated with homeostasis of the cell population. As remarked by Marciniak-Czochra et al. (2009), it is clear that convergence to a positive equilibrium is not feasible without regulation. In the case of two compartments, Getto et al. (2013) showed analytically that there exists a unique positive equilibrium in a certain region of the parameter space, while there is no positive equilibrium in the remaining region. Via a Lyapunov function it is proven that the positive equilibrium is globally stable under various regulation modes. Hence, Marciniak-Czochra et al. (2009) and Getto et al. (2013) found similar stability results, but they did not find destabilisation of the positive equilibrium (although Marciniak-Czochra et al. (2009) presented an example where the positive equilibrium is unstable on the whole existence region). These results are consistent with our findings in the sense that convergence to a positive equilibrium is possible when the stem cells population net growth rate is regulated.

Nakata et al. (2012) considered one unstructured progenitor compartment besides stem and mature cells, resulting in a three-compartment model. They proved that only in presence of the intermediate compartment the positive equilibrium can destabilise upon variation of parameters. Consistently with the analysis of Nakata et al. (2012), we have shown that adding a compartment between stem and mature cells can—depending on parameters—either preserve stability or allow for destabilisation. Such intermediate compartment is considered unstructured by Nakata et al. (2012), whereas it has a continuous maturity structure in the present paper. Comparing the stability boundaries of Nakata et al. (2012) with the ones found here, however, many substantial differences in shapes can be noted. It could be interesting to analyse these differences more precisely, in particular in relation with experimental data. Stiehl and Marciniak-Czochra (2011) and Nakata et al. (2012) showed that models with more than two unstructured progenitor compartments have, next to a zero and a positive equilibrium, also semi-trivial equilibria where the total amount of cells is positive, but where stem cells are absent. Apart from complicating the mathematical analysis, this feature may be questionable in terms of biological interpretation. As already observed by Doumic et al. (2011), models with a continuously-structured progenitor compartment do not allow for this type of semi-trivial equilibria and may therefore be more realistic in some biological contexts.

We remark that Doumic et al. (2011) studied a model similar to the one considered here, formulated as a transport equation. The paper contains results on boundedness of solutions, linearised stability, and persistence. Through formal linearisation (substituting exponential trial solutions) and numerical simulations, the authors found evidence for stability and for the destabilisation of the positive equilibrium via a Hopf bifurcation. In our stability analysis, we added a degree of reliability by quoting theorems that guarantee well-posedness and local correspondence of (in-)stability between original and linearised system, together with theorems that ensure that the model satisfies the necessary preconditions of the former theorems. Regarding the model ingredients, Doumic et al. (2011) focused mainly on a particular case motivated by the multi-compartment model,which is a special case of (8.2) with $$ g_0=k_1=0$$. Moreover, the progenitor production rate is not regulated by mature cells. Our results of stability on emergence hold for the more general model that incorporates a generic maturation rate *g*(*x*, *v*) and a progenitor production rate *d*(*x*, *v*) which may depend on mature cells.

One of the important results of Doumic et al. (2011) is the possibility of destabilisation of the positive equilibrium, which they accomplished by finding a parameter interval where destabilisation is possible, and by means of numerical examples. One of our aims here was to develop analytical and numerical methods for a quantification of the analysis, in particular the exact computation of destabilisation points and stability boundaries in parameter planes.

With analytical methods we have proven that the boundaries show interesting phenomena such as switches from boundedness to unboundedness in the stability region, and the appearance and disappearance of monotonicity upon variation of a third parameter, see Fig. 4. The presence of local minima corresponds to switches of the type stable-unstable-stable upon variation of a single parameter, as evident for instance from Fig. 4 (cases $\eta =0.99$ and $\eta =2.5$), when varying the parameter $\mu$.

The numerical computations show a wider range of potentially interesting phenomena. For instance, the type of regulation of the maturation rate by mature cells has significant impact: note the drastic differences in the stability boundaries in Figs. 6 and 7. The pseudospectral discretisation method allowed us to conduct the numerical analysis for the fully general model, although, with our specifications of the rates, adding maturity dependence in the maturation rate seemed to affect the boundaries mainly quantitatively rather than qualitatively, see Figs. 9 and 10 .

The proven features regarding the shapes of the stability boundaries could be a good starting point to understand possible biological mechanisms relating parameters at the cell level with behaviours observable at the cell population level. The numerical methods would then allow for a wider biological analysis: it would be interesting especially to exploit the flexibility of the pseudospectral approximation to conduct further analyses with parameter estimates coming from the experimental data.

We remark that we could find very few references among the modelling literature for a mathematical description of the maturation speed. One possible reason for this may be the fact that numerical methods being able to handle maturation rates dependent on maturity are not widespread, and that computation times significantly increase for such rates. A second possible reason is the biological state of the art, since cell maturation is not completely understood yet. We hope that the exploration contained in this paper, showing that the maturation rate strongly affects the stability of the positive equilibrium, can contribute to raise interest in such biological questions.

On the mathematical side, it could be interesting to investigate if explicit representations of stability boundaries can be computed algebraically for the case of a non-constant maturation rate, and whether qualitative changes with respect to the constant case can be shown. The numerical computations, together with the fact that qualitative changes appear already when varying the value of the constant maturation rate, suggest an affirmative answer.

Moreover, there is a clear analytical and numerical evidence that the destabilisation of the positive equilibrium occurs through a Hopf bifurcation. It could be interesting to prove the Hopf bifurcation analytically. As a “warm-up”, for the fixed-delay case one could check whether the Hopf bifurcation theorem by Diekmann et al. (1995) is applicable. For the state-dependent case, one could study related Hopf bifurcation theorems by Hu and Wu (2010). We refer to Adimy et al. (2010) for a Hopf bifurcation analysis in the context of an SD-DDE modelling cell maturation, which uses a result from Eichmann (2006). An ongoing project is the proof of existence of periodic solutions as fixed points of the next-state operators for the stem cell SD-DDE.

For a progenitor maturation rate that depends on maturity and mature cells, at present, pseudospectral methods seem to be the only method of analysis. This paper represents the first application of the method to a SD-DDE. This was possible by exploiting the uniform bound of the delay (3.5), and by applying the discretisation technique used for fixed-delay equations to the interval $[-h,0]$. We argued that the convergence results proven in the fixed-delay case remain true in this new context. We also showed that, for fixed-delay equations, the stability curves obtained with pseudospectral methods are indistinguishable from the ones obtained with the well-established software package dde-biftoolEngelborghs et al. (2002). This agreement supports numerically the convergence of the pseudospectral approximations of the stability and bifurcation properties of equilibria.

After the successful investigation of the stability of equilibria, it would be interesting to exploit the features of the software packages for ODE to push the analysis forward and investigate also the periodic solutions emerging from the Hopf bifurcations, together with their stability and bifurcation properties. We recall that the proof of convergence of the approximation of periodic solutions is still under investigation, but the numerical tests of Breda et al. (2016a, b) support the conjecture of spectral accuracy for equations with fixed delay. We remark also that, when the ODE describing the evolution of the structuring variable cannot be solved explicitly, like in the maturity-dependent cases considered in this paper, the computation times may be significant. An interesting future perspective is the application of the pseudospectral approximation technique to complex structured models of the type proposed by Diekmann et al. (2010), with a special attention to the efficiency and computation times.

Finally, we remark that, in the spirit of physiologically structured population models, it could be interesting to reformulate the present model as a Volterra functional equation coupled with a DDE, for which the principle of linearised stability was proven by Diekmann et al. (2007) and Diekmann and Gyllenberg (2012). One of the advantages of that formulation would be a larger set of admissible initial conditions.

## Proofs of Sections 3–6

### Proof of Theorem 3.1

Walther (2003) and Hartung et al. (2006) established that, if a functional *f* satisfies (S), then it is possible to define a *solution manifold* such that, for every initial condition belonging to the manifold, the initial value problem associated with the DDE induced by *f* admits a unique solution defined on a maximal interval. Moreover, the solutions define a differentiable semiflow, and a linear variational equation can be associated with the derivative. Getto and Waurick (2016) showed that, if (S) and (sLb) hold, the solutions are global, meaning that they are defined on $[-h,\infty )$. This proves the well-posedness statement. In Theorem 1.1 by Stumpf (2016) it was established that, again under condition (S), an equilibrium is locally asymptotically stable if all spectral values of the generator of the semigroup defined by the linear variational equation evaluated at equilibrium have negative real part, and unstable if there is such a value with positive real part (for the proofs it is referred to Theorem 3.6.1 of Hartung et al. (2006) for the stability criterion, and to Proposition 1.4 of Stumpf (2010) for the instability criterion). Hartung et al. (2006) and Stumpf (2016) established that these spectral values are given by the roots of the characteristic equation that is obtained by the familiar Ansatz of substituting exponential trial solutions into (3.1). Such a characteristic equation was expressed as (3.2) by Diekmann et al. (1995). This shows that also the statements on stability and instability hold and completes the proof. $\square$

The following result will be used to compute the characteristic equation. For the corresponding differentiability proofs, we refer to Getto and Waurick (2016).

### Lemma A.1

If (*w*, *v*) denotes an arbitrary equilibrium, we get

$$\begin{aligned}&D_1F_1(w,v)e_z =q(v), \quad D_2F_1(w,v)e_z=q'(v)w,\\&D_1F_2(w,v)e_z =\frac{\gamma (v)g(x_2,v)\mathop {}\!\mathrm {e}^{\int _0^{\tau (v)}(d-D_1g)(y(t,v),v)\mathop {}\!\mathrm {d}t}}{g(x_1,v)}\mathop {}\!\mathrm {e}^{-\tau (v)z}\\&D_2F_2(w,v)e_z =\frac{w\gamma (v)g(x_2,v)}{g(x_1,v)}\mathop {}\!\mathrm {e}^{\int _0^{\tau (v)}(d-D_1g)(y(t,v),v)\mathop {}\!\mathrm {d}t}\\&\qquad \Big \{\Big [\frac{\gamma '}{\gamma }(v)-\frac{D_2g}{g}(x_1,v)\Big ]\mathop {}\!\mathrm {e}^{-\tau (v)z}\\&\qquad +\frac{D_2g}{g}(x_2,v)+\int _0^{\tau (v)}\Big [D_2(d-D_1g)(y(t,v),v)\\&\qquad -D_2g(y(t,v),v)\;\Big (\frac{d-D_1g}{g}(x_1,v) \mathop {}\!\mathrm {e}^{-\int _t^{\tau (v)}D_1g(y(\theta ,v),v)\mathop {}\!\mathrm {d}\theta }\\&\qquad +\int _t^{\tau (v)}D_1(d-D_1g)(y(\sigma ,v),v)\mathop {}\!\mathrm {e}^{-\int _t^{\sigma }D_1g(y(\theta ,v),v)\mathop {}\!\mathrm {d}\theta }\mathop {}\!\mathrm {d}\sigma \Big )\Big ]\mathop {}\!\mathrm {e}^{-zt}\mathop {}\!\mathrm {d}t\Big \}-\mu . \end{aligned}$$

For the positive equilibrium, we additionally get

$$\begin{aligned}&D_1F_1(w,v)e_z=0, \;D_2F_1(w,v)e_z\\&\quad =\,\frac{q'(v)\mu vg(x_1,v)}{\gamma (v)g(x_2,v)} \mathop {}\!\mathrm {e}^{-\int _0^{\tau (v)}(d-D_1g)(y(t,v),v)\mathop {}\!\mathrm {d}t}, \\&D_2F_2(w,v)e_z=\widehat{k(v)}(z)+\mu v\Big [\frac{\gamma '}{\gamma }(v)-\frac{D_2g}{g}(x_1,v)\Big ]\mathop {}\!\mathrm {e}^{-\tau (v)z}\\&\quad +\,\mu \Big [v\frac{D_2g}{g}(x_2,v)-1\Big ]. \end{aligned}$$

with *k*(*v*) defined in (4.3).

### Proof of Lemma A.1

First, as proven in Propositions 1.9 and 1.11 by Getto and Waurick (2016), by differentiating (2.5) and (2.6) we can express

$$\begin{aligned} D_2y(t,v)\psi&=-\int _0^t \mathop {}\!\mathrm {e}^{-\int _\sigma ^t D_1g(y(\theta ,v),v)\mathop {}\!\mathrm {d}\theta }D_2g(y(\sigma ,v),v)\psi (-\sigma )\mathop {}\!\mathrm {d}\sigma ,\\ D\tau (v)\psi&=\frac{D_2y(\tau (v),v)\psi }{g(x_1,v)}\\&= -\frac{1}{g(x_1,v)}\int _0^{\tau (v)} \mathop {}\!\mathrm {e}^{-\int _t^{\tau (v)}D_1g(y(\theta ,v),v)\mathop {}\!\mathrm {d}\theta }\\&\quad D_2g(y(t,v),v)\psi (-t)\mathop {}\!\mathrm {d}t. \end{aligned}$$

This leads to

$$\begin{aligned}&\int _0^{\tau (v)}D_1(d-D_1g)(y(t,v),v)D_2y(t,v)\psi \mathop {}\!\mathrm {d}t \\&\quad =\, -\int _0^{\tau (v)}D_1(d-D_1g)(y(t,v),v) \int _0^t \mathop {}\!\mathrm {e}^{-\int _\sigma ^t D_1g(y(\theta ,v),v)\mathop {}\!\mathrm {d}\theta }\\&\quad D_2g(y(\sigma ,v),v)\psi (-\sigma )\mathop {}\!\mathrm {d}\sigma \mathop {}\!\mathrm {d}t \\&\quad =\,-\int _0^{\tau (v)}\int _t^{\tau (v)}D_1(d-D_1g)(y(\sigma ,v),v) \mathop {}\!\mathrm {e}^{-\int _t^\sigma D_1g(y(\theta ,v),v)\mathop {}\!\mathrm {d}\theta }\mathop {}\!\mathrm {d}\sigma \\&\quad D_2g(y(t,v),v)\psi (-t)\mathop {}\!\mathrm {d}t. \end{aligned}$$

We then get

$$\begin{aligned}&D_1F_1(w,v)\varphi =q(v)\varphi (0), \quad D_2F_1(w,v)\psi =wq'(v)\psi (0),\\&D_1F_2(w,v)\varphi =\frac{\gamma (v)g(x_2,v)\mathop {}\!\mathrm {e}^{\int _0^{\tau (v)}(d-D_1g)(y(t,v),v)\mathop {}\!\mathrm {d}t}}{g(x_1,v)}\varphi (-\tau (v)), \end{aligned}$$

and

$$\begin{aligned}&D_2F_2(w,v)\psi =w\mathop {}\!\mathrm {e}^{\int _0^{\tau (v)}(d-D_1g)(y(t,v),v)\mathop {}\!\mathrm {d}t}\\&\quad \Big \{\frac{g(x_2,v)}{g(x_1,v)}\gamma '(v)\psi (-\tau (v))+\frac{\gamma (v)}{g(x_1,v)}D_2g(x_2,v)\psi (0)\\&\qquad -\gamma (v)\frac{g(x_2,v)}{g^2(x_1,v)}D_2g(x_1,v)\psi (-\tau (v)) +\frac{\gamma (v)g(x_2,v)}{g(x_1,v)}\\&\qquad \Big [(d-D_1g)(x_1,v)D\tau (v)\psi \\&\qquad +\int _0^{\tau (v)}D_1(d-D_1g)(y(t,v),v)D_2y(t,v)\psi \\&\qquad +D_2(d-D_1g)(y(t,v),v)\psi (-t)\mathop {}\!\mathrm {d}t\Big ]\Big \}-\mu \psi (0)\\&\quad =\frac{w}{g(x_1,v)}\mathop {}\!\mathrm {e}^{\int _0^{\tau (v)}(d-D_1g)(y(t,v),v)\mathop {}\!\mathrm {d}t}\\&\qquad \Big \{g(x_2,v)\Big [\gamma '(v)-\frac{\gamma (v)}{g(x_1,v)}D_2g(x_1,v)\Big ]\psi (-\tau (v))\\&\qquad +\gamma (v)D_2g(x_2,v)\psi (0) +\gamma (v)g(x_2,v)\\&\qquad \int _0^{\tau (v)}\Big [D_2(d-D_1g)(y(t,v),v)\\&\qquad -\frac{d-D_1g}{g}(x_1,v) \mathop {}\!\mathrm {e}^{-\int _t^{\tau (v)}D_1g(y(\theta ,v),v)\mathop {}\!\mathrm {d}\theta }D_2g(y(t,v),v)\\&\qquad -\int _t^{\tau (v)}D_1(d-D_1g)(y(\sigma ,v),v)\mathop {}\!\mathrm {e}^{-\int _t^{\sigma }D_1g(y(\theta ,v),v)\mathop {}\!\mathrm {d}\theta }\mathop {}\!\mathrm {d}\sigma \\&\qquad D_2g(y(t,v),v)\Big ]\psi (-t)\mathop {}\!\mathrm {d}t\Big \}-\mu \psi (0). \end{aligned}$$

Evaluation at $e_z$ yields the result for an arbitrary equilibrium. Additionally, using the conditions for the positive equilibrium, we get that the first derivative becomes zero, and we can eliminate *w* and substitute the expression into $D_2F_1$ and $D_2F_2$. With the definitions of *k*(*v*) given in (4.3), the thesis follows. $\square$

### Proof of Lemma 4.1

The characteristic equation for the trivial equilibrium follows from the computation

$$\begin{aligned} \det (zI-DF(0)e_z)=\det \left( \begin{array}{cc} z-q(0)&{}0\\ -D_1F_2(0)e_z&{}z+\mu \end{array} \right) . \end{aligned}$$

Let now (*w*, *v*) denote the positive equilibrium. Then, using the previous lemma, we can write

$$\begin{aligned} D_2F_2(w,v)e_z=a(v)+b(v)\mathop {}\!\mathrm {e}^{-\tau (v)z}+\widehat{k(v)}(z), \end{aligned}$$

where

$$\begin{aligned} a(v):=\mu \left( v\frac{D_2g}{g}(x_2,v)-1\right) , \quad b(v):=\mu v\left( \frac{\gamma '}{\gamma }(v)-\frac{D_2g}{g}(x_1,v)\right) . \end{aligned}$$

Then, by using the expressions obtained in Lemma A.1, the expression for the characteristic equation for the positive equilibrium follows from the computation

$$\begin{aligned} \det (zI-DF(w,v)e_z)= & {} z\left[ z-(a(v)+b(v)\mathop {}\!\mathrm {e}^{-\tau (v)z}\right. \\&\left. +\,\widehat{k(v)}(z))\right] -q'(v)\mu v \mathop {}\!\mathrm {e}^{-\tau (v)z}\\= & {} -z\widehat{k(v)}(z)+\mathop {}\!\mathrm {e}^{-\tau (v)z}\left[ -zb(v)-q'(v)\mu v\right] \\&+\,z^2-a(v)z. \end{aligned}$$

$$\square $$

### Proof of Lemma 4.3

It is trivial to see that $\chi (0,0)=0$. To conclude that $\chi$ is analytic, one can for instance show that $z\mapsto \chi (v,z)$ satisfies the Cauchy–Riemann equations. Moreover $D_1\chi (0,0)=q'(0)\mu$ and $D_2\chi (0,0)=\widehat{k(0)}(0)-\mu =-\mu$, since $k(0)=0$. Since $z\mapsto \chi (v,z)$ is analytic with $D_2\chi (0,0)\ne 0$, the implicit function theorem implies the statements with

$$\begin{aligned}&z'(0)=\frac{-D_1\chi }{D_2\chi }(0,0)=q'(0)<0. \end{aligned}$$

### Proof of Lemma 4.4

We can rewrite (4.2) as

A.1 $$\begin{aligned} z\widehat{k(v)}(z)+\mathop {}\!\mathrm {e}^{-\tau (v)z}(a_1z+a_2)-z^2+a_3z=0, \end{aligned}$$

where, here and in the following, the $a_i$ are *z*-independent quantities with obvious definitions. By continuity of $t\mapsto k(v)(t)$ and since $\mathop {}\!\mathrm {Re}\,z>0$, we have (independently of the shape of *k*(*v*)) that

$$\begin{aligned} |\widehat{k(v)}(z)|\le \tau (v) \max _{t\in [0,\tau (v)]} |k(v)(t)| \end{aligned}$$

and $$|\mathop {}\!\mathrm {e}^{-\tau (v)z}|\le 1$$, hence

$$\begin{aligned} \big |z\widehat{k(v)}(z)+\mathop {}\!\mathrm {e}^{-\tau (v)z}(a_1z+a_2)\big |\le |z|a_4+a_5 \end{aligned}$$

for some nonnegative $a_4$ and $a_5$. Since $|z^2-a_3z|\ge |z|^2-a_6|z|$ for nonnegative $a_6$, it follows from (A.1) that

$$\begin{aligned} |z|a_4+a_5\ge |z|^2-a_6|z|. \end{aligned}$$

This implies $|z|^2\le a_7|z|+a_5$, thus $|z|\le a_7+\frac{a_5}{|z|}$ for nonnegative $a_7$. Hence $|z|\le K$ with $K:=\max \{1,a_5+a_7\}$. $\square$

### Proof of Lemma 4.5

The representation (4.4) follows from Lemma 4.1 if we show that *k*(*v*), and therefore $\widehat{k(v)}$, change as stated. The latter follows by combining the two identities

A.2 $$\begin{aligned} \int _t^{\tau (v)}&D_1d(y(\sigma ,v),v)\mathop {}\!\mathrm {d}\sigma \nonumber \\&=\frac{1}{g(v)}(d(y(t,v),v)-d(x_1,v)), \end{aligned}$$

A.3 $$\begin{aligned} k(v)(t)&=\mu v\Big [D_2d(y(t,v),v)-d(x_1,v)\frac{g'}{g}(v) \nonumber \\&\quad -\,g'(v)\int _t^{\tau (v)}D_1d(y(\sigma ,v),v)\mathop {}\!\mathrm {d}\sigma \Big ]. \end{aligned}$$

Then (4.6) is obvious. Using the specifications, (4.7) follows in a straightforward way from (4.6), since

$$\begin{aligned}&\gamma '(v)=2[-s'(v)d_w(v)+(1-s(v))d_w'(v)],\\&\frac{\gamma '(v)}{\gamma (v)}=\frac{d_w'(v)}{d_w(v)}-\frac{s'(v)}{1-s(v)},\\&q'(v)=2s'(v)d_w(v)+d_w'(v)[2s(v)-1]. \end{aligned}$$

Using the new specifications, the characteristic Eq. (4.7) becomes

$$\begin{aligned} \frac{d_w'}{d_w}(v)\mu v\big [z+d_w(v)(2a-1)\big ]\mathop {}\!\mathrm {e}^{-z\tau }-z^2-\mu z=0. \end{aligned}$$

If we use the equilibrium condition $(2a-1)d_w(v)-\mu _w=0$, we get (4.8). Finally, for the specification of $d_w$ and using the equilibrium conditions we get

$$\begin{aligned} \frac{d_w'(v)}{d_w(v)} v=\frac{\mu _w}{(2a-1)p}-1, \end{aligned}$$

with which (4.9) follows from (4.8). $\square$

### Proof of Lemma 5.2

(a) We prove only some statements for $f_\eta$ for $\eta <1$, since the proofs of the remaining statements do not use new techniques. One has $f_\eta (2k\pi )=-2k\pi \le 0$, $f_\eta (\pi /2+2k\pi )=\eta$, $f_\eta '(\omega )=(\eta -1)\cos \omega +\omega \sin \omega$, $f_\eta '(2k\pi )=\eta -1<0$, $f_\eta '(\pi /2+2k\pi )=\pi /2+2k\pi >0$ and $f''_\eta (\omega )=-(\eta -2)\sin \omega +\omega \cos \omega >0$ in between, since both of its addends are positive. Thus $f'_\eta$ increases from a negative to a positive value. Hence, $f_\eta$ first decreases from a nonpositive value, then increases to a positive value. Thus there exists a unique zero in every $(0,\pi /2)+2k\pi$. (b) is an obvious corollary from (a).

(c) On $[\omega _1,\pi /2]$ the function $f_\eta '$ is positive by the arguments used in the proof of (a). On $[\pi /2,\omega _2]$ it is positive since both addends of the expression, see again proof of (a), are positive on $[\pi /2,\pi ]$. $\square$

### Proof of Lemma 5.3

In $(m,r,0,\omega )$, one computes

$$\begin{aligned}&\partial _1 H_1=\partial _m H_1=r(\eta \cos \omega +\omega \sin \omega ),\\&\partial _2 H_1=\partial _r H_1=m(\eta \cos \omega +\omega \sin \omega ),\\&\partial _1 H_2=\partial _m H_2=\omega +r(\omega \cos \omega -\eta \sin \omega ), \\&\partial _2 H_2=\partial _r H_2=m(\omega \cos \omega -\eta \sin \omega ), \end{aligned}$$

and from this the statement follows. $\square$

In the next two proofs, we do not denote subindices of $f_\eta$ and $g_\eta$.

### Proof of Lemma 5.4

Let first $\eta \ge 1$. One has $m(\omega )=\frac{\omega f(\omega )}{g(\omega )}$ and

$$\begin{aligned} f'(\omega )=g(\omega )-\cos \omega , \quad g'(\omega )=\sin \omega -f(\omega ). \end{aligned}$$

Then,

$$\begin{aligned} {{\,\mathrm{sgn}\,}}m'(\omega )&={{\,\mathrm{sgn}\,}}[f(\omega )+\omega f'(\omega )]g(\omega )-\omega f(\omega )g'(\omega )\\&={{\,\mathrm{sgn}\,}}f(\omega )g(\omega )+\omega g^2(\omega )+\omega f^2(\omega )\\&\quad -\,\omega g(\omega )\cos \omega -\omega f(\omega )\sin \omega ={{\,\mathrm{sgn}\,}}h(\omega ) \end{aligned}$$

for *h* defined in an obvious way. Note that the first three terms have positive sign. On $(0,\pi /2]$ we have

$$\begin{aligned} f(\omega )g(\omega )-\omega f(\omega )\sin \omega= & {} f(\omega )(g(\omega )-\omega \sin \omega )\\= & {} f(\omega )\eta \cos \omega>0,\\ \omega g^2(\omega ) -\omega g(\omega )\cos \omega= & {} \omega g(\omega )(g(\omega )-\cos \omega ) \\= & {} \omega g(\omega )[(\eta -1)\cos \omega +\omega \sin \omega ]>0. \end{aligned}$$

Thus ${{\,\mathrm{sgn}\,}}m'(\omega )=1$ on $(0,\pi /2]$. Similarly, on $[\pi /2,\omega _1]$ we have

$$\begin{aligned} \omega f^2(\omega )-\omega f(\omega )\sin \omega>0,\quad f(\omega )g(\omega )-\omega g(\omega )\cos \omega >0. \end{aligned}$$

Thus ${{\,\mathrm{sgn}\,}}m'(\omega )=1$ also on $[\pi /2,\omega _1]$, hence on $(0,\omega _1)$.

Let now $\eta <1$. Then $f(\omega _1)=0$ implies that

$$\begin{aligned} h(\omega _1)=\omega _1g(\omega _1)(g(\omega _1)-\cos \omega _1)= \omega _1g(\omega _1)f'(\omega _1)>0. \end{aligned}$$

Now, not denoting the $\omega$-argument where convenient,

$$\begin{aligned} h'= & {} f'g+fg'+g^2+2\omega gg'+f^2+2\omega ff'\\&-\,g\cos -\omega g'\cos +\omega g\sin -f\sin -\omega f'\sin -\omega f \cos \\= & {} 2g(g-\cos )+2\omega (g\sin -f\cos )=2gf'+2\omega ^2>0. \end{aligned}$$

Thus $h(\omega )>0$ on $(\omega _1,\omega _2)$. Hence the statement follows. $\square$

### Proof of Lemma 5.5

By Lemma 5.4 it suffices to show the statement for *r* as a function of $\omega$. Using that $r(\omega )=\frac{\omega }{f(\omega )}$, we get ${{\,\mathrm{sgn}\,}}r'(\omega )={{\,\mathrm{sgn}\,}}h(\omega )$, where

$$\begin{aligned} h(\omega ):= & {} f(\omega )-\omega f'(\omega )= (\eta -\omega ^2)\sin \omega -\omega \eta \cos \omega =\eta \sin \omega -\omega g(\omega ). \end{aligned}$$

If the sign of a function is not obvious we consider the derivative. We compute

$$\begin{aligned} h'(\omega )= & {} -\omega [(2-\eta )\sin \omega +\omega \cos \omega ], \\ h''(\omega )= & {} (\omega ^2+\eta -2)\sin \omega +\omega (\eta -4)\cos \omega ,\\ h'''(\omega )= & {} \omega (6-\eta )\sin \omega +(\omega ^2+2\eta -6)\cos \omega ,\\ h^{(4)}(\omega )= & {} (12-3\eta -\omega ^2)\sin \omega +\omega (8-\eta )\cos \omega . \end{aligned}$$

Now, let first $\eta \in [1,2]$. Then, $h'|_{(0,\frac{\pi }{2})}<0$ since $\eta \le 2$ and, since moreover $h(0)=0$, we have $h|_{(0,\frac{\pi }{2})}<0$. Next, using $g(\omega _1)=0$, we get

$$\begin{aligned} h(\omega _1)= & {} \eta \sin \omega _1-\omega _1g(\omega _1)=\eta \sin \omega _1>0,\\ h'(\omega _1)= & {} [-(\eta -1)^2+1-\omega _1^2]\cos \omega _1>0,\;\mathrm{since}\;\omega _1\in (\frac{\pi }{2},\pi ),\\ h''(\omega )> & {} 0\;\mathrm{on}\;(\frac{\pi }{2},\pi ),\;\mathrm{since}\; \eta \in [1,2]. \end{aligned}$$

Hence $h'$ increases from negative to positive on $[\frac{\pi }{2},\omega _1]$. Thus *h* decreases from zero in zero to a minimum, which lies to the right of $\frac{\pi }{2}$, then increases to a positive value in $\omega _1$. Hence, *r* as a function of $\omega$ decreases from a positive value to a minimum, also to the right of $\frac{\pi }{2}$, then increases. This implies the statement for $\eta \in [1,2]$.

Let now $\eta <1$. Then $h(\omega _1)=-\omega _1f'(\omega _1)<0$. Since moreover $h'<0$ on $(\omega _1,\frac{\pi }{2}]$ we have $h<0$ on $[\omega _1,\frac{\pi }{2}]$. Next, $h(\omega _2)=\eta \sin \omega _2-\omega _2g(\omega _2)=\eta \sin \omega _2>0$. Now $h'(\frac{\pi }{2})<0$ and, using $g(\omega _2)=0$, we get

$$\begin{aligned} h'(\omega _2)= & {} -\omega _2\left( 2-\eta -\frac{\omega _2^2}{\eta }\right) \sin \omega _2>0, \end{aligned}$$

as the term in brackets is negative since $\omega _2^2\ge \frac{\pi ^2}{4}>1\ge \eta (2-\eta )$. Moreover $h''>0$ on $(\frac{\pi }{2},\omega _2)$. Hence, $h'$ increases from negative to positive on $(\frac{\pi }{2},\omega _2)$. Thus, *h* decreases from negative then increases to positive and the zero lies in $(\frac{\pi }{2}, \omega _2)$. The statement for *r* for $\eta <1$ follows.

Now consider $\eta \in (2,3)$. We first consider $[0,\frac{\pi }{2}]$. Then $h'''(0)=2\eta -6<0$. Moreover $12-3\eta -\omega ^2>3-\frac{\pi ^2}{4}>0$, hence $h^{(4)}>0$ on $(0,\frac{\pi }{2})$. Now $h'''(\frac{\pi }{2})=\frac{\pi }{2}(6-\eta )>0$. Thus $h'''$ increases from negative to positive. Since moreover $h''(0)=0$ and $h''(\frac{\pi }{2})=\frac{\pi ^2}{4}+\eta -2>0$ this implies that $h''$ decreases from zero, then increases to positive. Since $h'(\frac{\pi }{2})=-\frac{\pi }{2}(2-\eta )>0$ so does $h'$. As $h(\frac{\pi }{2})=\eta -\frac{\pi ^2}{4}$ the function *h* decreases from zero, then increases, to negative if $\eta <\frac{\pi ^2}{4}$, to zero, if $\eta =\frac{\pi ^2}{4}$ and to positive if $\eta >\frac{\pi ^2}{4}$. We next consider $[\frac{\pi }{2},\pi ]$. Then $h'>0$. Now, since $h(\omega _1)>0$ the function *h* increases on $(\frac{\pi }{2},\omega _1)$ from positive, if $\eta >\frac{\pi ^2}{4}$, from zero, if $\eta =\frac{\pi ^2}{4}$, and from negative to positive, if $\eta <\frac{\pi ^2}{4}$. This implies the statement for $\eta \in (2,3)$.

We now first consider $\eta \ge 4$. Then $h''>0$ on $(0,\frac{\pi }{2}]$. Thus $h'$ increases from zero in zero to a positive value in $\frac{\pi }{2}$. Hence, so does *h*. Let now $\eta \in [3,4]$. Then $h'''>0$ on $(0,\frac{\pi }{2}]$. It follows that $h''$, thus $h'$, hence *h* is positive on $(0,\frac{\pi }{2}]$. Now consider all $\eta \ge 3$. Then $h'>0$ on $[\frac{\pi }{2},\pi ]$. Hence *h* remains positive on $[\frac{\pi }{2},\omega _1)$, thus is positive on $(0,\omega _1]$, hence *r* increases. $\square$

### Proof of Lemma 6.1

Assumption (G3) in Sect. 3 ensures that $\varPhi _v$ and $T_u$ are monotonically increasing and hence injective. From Proposition 1.9(a) by Getto and Waurick (2016), there exists a unique $\tau =\tau (v_0)\in (-h,0)$ such that $\varPhi _v(-\tau )=-\delta$, hence $[-\delta ,0]\subseteq \varPhi _v([-h,0])$ and $\varPhi _v$ is surjective on $[-\delta ,+\infty )$. Moreover, $\varPhi _v^{-1}$ satisfies

A.4 $$\begin{aligned} (\varPhi _v^{-1})'(\phi ) = \frac{1}{g(v(\varPhi _v^{-1}(\phi )))}, \quad \phi \ge -\delta , \end{aligned}$$

hence $\varPhi _v^{-1}$ is continuously differentiable for $\phi \ge -\delta$.

From assumption (G3), we can bound

$$\begin{aligned} -\tau _{\zeta } = T_{\zeta }(-\delta ) \ge -\frac{\delta }{\varepsilon } \ge -\frac{b}{K} = - h, \end{aligned}$$

hence $T_{\zeta }([-\delta ,0])=[-\tau _{\zeta },0] \subseteq [-h,0]$ and $T_{\zeta }$ is surjective onto $[-\tau _{\zeta },0]$. This implies that $T_u$ is invertible with $T_u^{-1}:[-\tau _{\zeta },+\infty ) \rightarrow {\mathbb {R}}$ satisfying

$$\begin{aligned} (T_u^{-1})'(t) = g(u(T_u^{-1}(t))), \quad t \ge -\tau _{\zeta }. \end{aligned}$$

$$\square $$

### Proof of Theorem 6.2

Let (*w*, *v*) be a solution of (2.7) and (6.1) with initial condition (3.6), and $(\omega ,u)$ be defined by (6.8). From Lemma 6.1 we have that $\varPhi _v^{-1} \in C^1([-\delta ,+\infty ),{\mathbb {R}})$, hence $(\omega ,u)$ is continuously differentiable on $[-\delta ,+\infty )$. Moreover, (6.7) holds true by definition. Thus, we only need to prove that $(\omega ,u)$ satisfies (6.5) and (6.6). From (6.3) we conclude that, for all $t> 0$,

A.5 $$\begin{aligned} \varPhi _v(t)-\varPhi _v(t-\tau (v_t)) = \delta . \end{aligned}$$

Hence, for all $t>0$ we can rewrite the discrete delay terms as

A.6 $$\begin{aligned} w(t-\tau (v_t))&= \omega (\varPhi _v(t-\tau (v_t))) = \omega (\varPhi _v(t)-\delta ), \end{aligned}$$

A.7 $$\begin{aligned} v(t-\tau (v_t))&= u(\varPhi _v(t-\tau (v_t))) = u(\varPhi _v(t)-\delta ). \end{aligned}$$

Consider now the distributed delay term in (6.1), that we rewrite as

A.8 $$\begin{aligned} \int _0^{\tau (v_t)} d(y(s,v_t),v(t-s))\mathop {}\!\mathrm {d}s&= \int _{t-\tau (v_t)}^{t} d(y(t-\sigma ,v_t),v(\sigma ))\mathop {}\!\mathrm {d}\sigma . \end{aligned}$$

From (6.2), using the change of variable $\phi =\varPhi _v(\theta )$ and relation (A.4), we can write

$$\begin{aligned} y(t-\sigma ,v_t)&= x_2 - \int _{\sigma }^t g(v(\theta ))\mathop {}\!\mathrm {d}\theta \\&= x_2 - \int _{\sigma }^t g(u(\varPhi _v(\theta )))\mathop {}\!\mathrm {d}\theta \\&= x_2 - \int _{\varPhi _v(\sigma )}^{\varPhi _v(t)} \frac{g(u(\phi ))}{g(u(\phi ))}\mathop {}\!\mathrm {d}\phi \\&= x_2 - \left( \varPhi _v(t)-\varPhi _v(\sigma )\right) . \end{aligned}$$

Substituting into (A.8), we get

A.9 $$\begin{aligned}&\int _0^{\tau (v_t)} d(y(s,v_t),v(t-s))\mathop {}\!\mathrm {d}s\nonumber \\&= \int _{t-\tau (v_t)}^t d(x_2-(\varPhi _v(t)-\varPhi _v(\sigma )),u(\varPhi _v(\sigma )))\mathop {}\!\mathrm {d}\sigma \nonumber \\&= \int _{\varPhi _v(t-\tau (v_t))}^{\varPhi _v(t)} \frac{d(x_2-(\varPhi _v(t)-\phi ),u(\phi ))}{g(u(\phi ))} \mathop {}\!\mathrm {d}\phi \nonumber \\&= \int _0^{\delta } \frac{d(x_2-s,u(\varPhi _v(t)-s))}{g(u(\varPhi _v(t)-s))}\mathop {}\!\mathrm {d}s, \end{aligned}$$

where the last equality follows from (A.5). Finally, by applying the chain rule, we get

$$\begin{aligned} \omega '(\phi )&= w'(\varPhi ^{-1}(\phi )) (\varPhi ^{-1}_v)'(\phi ) = w'(\varPhi ^{-1}(\phi )) \frac{1}{g(u(\phi ))} \\ u'(\phi )&= v'(\varPhi ^{-1}(\phi )) (\varPhi ^{-1}_v)'(\phi ) = w'(\varPhi ^{-1}(\phi )) \frac{1}{g(u(\phi ))}. \end{aligned}$$

By using (2.7) and (6.1) with $t=\varPhi _v^{-1}(\phi )$ and substituting relations (A.6), (A.7) and (A.9), we conclude that $(\omega ,u)$ satisfies (6.5) and (6.6) for all $\phi > 0$.

Vice versa, let $(\omega ,u)$ be a solution of (6.5)–(6.7). From Lemma 6.1 it follows that $T_u^{-1}\in C^1([-\tau _{\zeta },+\infty ),{\mathbb {R}})$. Let $\varphi ,\psi$ be defined on $[-h,0]$ and satisfying (6.9), and let (*w*, *v*) be defined by (6.10) and extended such that $w_0=\varphi$, $v_0=\psi$. By definition, (*w*, *v*) is continuously differentiable on $[-\delta ,+\infty )$ and satisfies (3.6). We now show that (*w*, *v*) satisfies (2.7) and (6.1). Note that $T_u=\varPhi _v^{-1}$, since $\varPhi _v^{-1}(0)=T_u(0)=0$ and they have the same derivative for $\phi \ge 0$. Let $\tau =\tau (\psi )$ be defined implicitly by (6.3). For every $t>0$, $\tau (v_t)$ satisfies (A.5), and hence (A.6) and (A.7) hold true. The distributed delay term can be transformed analogously as before to obtain the identity (A.9). Finally, by applying the chain rule, we have

$$\begin{aligned} w'(t)&= \omega '(T_u^{-1}(t)) (T_u^{-1})'(t) = \omega '(T_u^{-1}(t)) g(v(t)) \\ v'(t)&= u'(T_u^{-1}(t)) (T_u^{-1})'(t) = u'(T_u^{-1}(t)) g(v(t)), \end{aligned}$$

and we conclude that (*w*, *v*) satisfies (2.7) and (6.1) for all $t>0$. $\square$

### Proof of Lemma 6.4

Lemma A.1 and Eqs. (A.2) and (A.3) imply that

A.10 $$\begin{aligned}&D_1F_1(w,v)e_z=0, \quad D_2F_1(w,v)e_z=\frac{q'(v)\mu v}{\gamma (v)} \mathop {}\!\mathrm {e}^{-\int _0^{\tau (v)}d(y(t,v),v)\mathop {}\!\mathrm {d}t}, \nonumber \\&D_1F_2(w,v)e_z=\gamma (v)\mathop {}\!\mathrm {e}^{\int _0^{\tau (v)}d(y(t,v),v)\mathop {}\!\mathrm {d}t}\mathop {}\!\mathrm {e}^{-\tau (v)z}, \nonumber \\&D_2F_2(w,v)e_z=\widehat{k(v)}(z)+\mu v\left( \frac{\gamma '}{\gamma }-\frac{g'}{g}\right) (v) \mathop {}\!\mathrm {e}^{-\tau (v)z}+\mu \left( v\frac{g'}{g}(v)-1\right) , \end{aligned}$$

with *k*(*v*) as in (4.5). We show only that the respective last entries agree, i.e., that

A.11 $$\begin{aligned} D_2G_2(w,v)e_{kz}=kD_2F_2(w,v)e_z, \end{aligned}$$

since the techniques for the remaining elements are less complex. First, one has

$$\begin{aligned} D_2G_2(w,v)\psi= & {} \frac{w\mathop {}\!\mathrm {e}^{\int _0^{\delta }\frac{d(x_2-s,v)}{g(v)}\mathop {}\!\mathrm {d}s}}{g(v)} \Big \{ \Big (\gamma '-\frac{g'\gamma }{g}\Big )(v)\psi (-\delta )\\&+\, \frac{\gamma }{g}(v)\int _0^\delta \Big [D_2d(x_2-s,v) -d(x_2-s,v)\frac{g'}{g}(v)\Big ]\psi (-s)\mathop {}\!\mathrm {d}s\Big \}\\&-\,\mu \Big (\frac{1}{g(v)}-v\frac{g'}{g^2}(v)\Big )\psi (0). \end{aligned}$$

Using the equilibrium condition $\mu v=\gamma (v)w\mathop {}\!\mathrm {e}^{\int _0^\delta \frac{d(x_2-t,v)}{g(v)}\mathop {}\!\mathrm {d}t}$, we get

$$\begin{aligned} D_2G_2(w,v)\psi= & {} \frac{\mu }{g(v)} \Big \{ v\Big [\big (\frac{\gamma '}{\gamma }-\frac{g'}{g}\big )(v)\psi (-\delta )\\&+\,\frac{1}{g(v)}\int _0^\delta \Big (D_2d(x_2-t,v) -d(x_2-t,v)\frac{g'}{g}(v)\Big )\psi (-t)\mathop {}\!\mathrm {d}t \Big ]\\&-\,\big (1-\frac{vg'}{g}(v)\big )\psi (0)\Big \}. \end{aligned}$$

Hence,

$$\begin{aligned} D_2G_2(w,v)e_{kz}=&\frac{\mu }{g(v)} \Big \{ \frac{v}{g(v)}\int _0^\delta \Big [D_2d(x_2-t,v)-d(x_2-t,v)\frac{g'}{g}(v)\Big ]\mathop {}\!\mathrm {e}^{-\frac{zt}{g(v)}}\mathop {}\!\mathrm {d}t \\&+\,\mu v\big (\frac{\gamma '}{\gamma }-\frac{g'}{g}\big )(v)\mathop {}\!\mathrm {e}^{-\frac{\delta z}{g(v)}}+ v\frac{g'}{g}(v)-1\Big \}. \end{aligned}$$

Comparing this expression with (A.10), it is easy to see that (A.11) holds. $\square$
